# Brief evidence-based interventions for universal child health services: a restricted evidence assessment of the literature

**DOI:** 10.1186/s12889-020-09104-7

**Published:** 2020-06-24

**Authors:** James J. Newham, Karen McLean, Samuel Ginja, Lisa Hurt, Carly Molloy, Raghu Lingam, Sharon Goldfeld

**Affiliations:** 1 Faculty of Health and Life Sciences, Northumbria University, Newcastle upon Tyne, NE1 8ST UK; 2 Murdoch Children’s Research Institute, University of Melbourne, Melbourne, Australia; 3 School of Psychology, Ulster University, Coleraine, UK; 4 Division of Population Medicine, Cardiff University School of Medicine, Cardiff, UK; 5 School of Women’s & Children’s Health, University of New South Wales, Randwick, Australia

**Keywords:** Child public health, Mental health, Sleep, Infant, Emotional and social wellbeing, Home learning improvement, Rapid evidence assessment

## Abstract

**Background:**

Universal child health services (UCHS) provide an important pragmatic platform for the delivery of universal and targeted interventions to support families and optimize child health outcomes. We aimed to identify brief, evidence-based interventions for common health and developmental problems that could be potentially implemented in UCHS.

**Methods:**

A restricted evidence assessment (REA) of electronic databases and grey literature was undertaken covering January 2006 to August 2019. Studies were eligible if (i) outcomes related to one or more of four areas: child social and emotional wellbeing (SEWB), infant sleep, home learning environment or parent mental health, (ii) a comparison group was used, (iii) universal or targeted intervention were delivered in non-tertiary settings, (iv) interventions did not last more than 4 sessions, and (v) children were aged between 2 weeks postpartum and 5 years at baseline.

**Results:**

Seventeen studies met the eligibility criteria. Of these, three interventions could possibly be implemented at scale within UCHS platforms: (1) a universal child behavioural intervention which did not affect its primary outcome of infant sleep but improved parental mental health, (2) a universal screening programme which improved maternal mental health, and (3) a targeted child behavioural intervention which improved parent-reported infant sleep problems and parental mental health. Key lessons learnt include: (1) Interventions should impart the maximal amount of information within an initial session with future sessions reinforcing key messages, (2) Interventions should see the family as a holistic unit by considering the needs of parents with an emphasis on identification, triage and referral, and (3) Brief interventions may be more acceptable for stigmatized topics, but still entail considerable barriers that deter the most vulnerable.

**Conclusions:**

Delivery and evaluation of brief evidence-based interventions from a UCHS could lead to improved maternal and child health outcomes through a more responsive and equitable service. We recommend three interventions that meet our criteria of “best bet” interventions.

## Background

There is now strong evidence that the early years of childhood, especially the first 1000 days from conception, impacts the long-term health, social and economic wellbeing of the individual across their life course [1–3]. Children who experience adversity in early childhood (e.g. poverty, parent mental illness, child abuse) are not only at increased risk of developmental delay [4, 5], but they are also at increased risk of poor health outcomes in later life [6]. Globally, the high prevalence of common health and developmental problems in families is associated with increasing social disadvantage [7]. Prevention of these problems, known as ‘millennial morbidities’, is increasingly seen as critical to addressing inequity and the future human capital of countries [8, 9]. Inequity is commonly seen as the presence of systematic and potentially remediable differences among population groups [10] and, as intervening in early life is the most cost-effective time to influence the health of an individual across the life course [11], it makes sense that universal child health services (UCHS) around the world are best placed to provide equitable and effective care. UCHS are a highly valued and critical part of the health system in most high-income countries (HIC), and delivered with remarkable similarity by nurses, health visitors and/or pediatricians [12]. Most services consistently provide a platform for early identification and referral for health and developmental problems, support for at-risk families, and health and developmental promotion.

While UCHS provide a potential platform for the delivery of evidence-based interventions, there are scant details regarding which interventions might be effective, or how to implement them [12]. The United States Institute of Medicine [13] put forward a comprehensive framework to classify public health prevention. Universal prevention is defined as those interventions that are aimed to a whole population group that have not been identified by increased risk, with the aim of reducing the incidence of problems, maladaptive behaviours or disorders before they manifest. Targeted prevention can be divided into two distinct types; selective and indicated. Selective interventions are aimed at individuals or subgroups who are at greater risks of adverse outcomes as evidenced by biological, psychological or social risk factors (e.g. poverty, ethnicity). Indicated interventions are aimed at individuals with pre-existing symptoms or pre-clinical diagnoses for adverse outcomes but who do not meet diagnostic criteria (e.g. patients with pre-gestational diabetes). It remains unclear which of these approaches is best to address millennial morbidities; or whether a combination is best that is modelled on proportionate universalism, an approach that involves the provision of a universal service to an entire population with a scale and intensity proportionate to the level of disadvantage and need [14].

Irrespective of whether a universal or targeted approach is taken, adoption of any intervention needs to be balanced against existing resources and its capacity to be implemented within existing infrastructures. Interventions delivered in a brief format could theoretically be more feasible and less costly to deliver by diverting families from more expensive and intensive referral services; simultaneously maximising the utility of already funded UCHS platforms. Furthermore, parents may be reluctant to engage with services from perceiving them as time consuming, disruptive and too overwhelming [15]. Brief interventions target a symptom or behavior by providing clients with tools to change basic attitudes and manage underlying problems for specific behavioral change [16]. As such there is a need to develop and implement intervention services in the early years that can be effectively delivered in as few sessions as possible to help improve engagement.

Given the dearth of evidence regarding brief interventions we aimed to identify universal and targeted ‘best bet’ evidence-based interventions that could be delivered in a brief number of sessions to positively affect parental and child health, wellbeing and development. Utilising the Rapid Evidence Assessment (REA) approach [17], we undertook a series of reviews related to four priority areas with increasing rates of global prevalence, and are a mixture of problems and protective factors that impact on the long-term health and wellbeing of children: (i) child social and emotional wellbeing (Child SEWB), (ii) infant sleep disorders, (iii) home learning environment, and (iv) parental mental health. This REA was conducted to provide an overview of the evidence relating to several outcomes. As such, data regarding effectiveness, acceptability, bias, and implementation were compared and interpreted across studies by authors to inform the identification of ‘best bet’ interventions and for the testing and implementation of brief interventions to guide commissioners, service providers, and evaluators. We hypothesise that brief interventions would be more acceptable to both families and healthcare practitioners as they may be easier to attend for those with child-caring responsibilities and entail less resources to deliver.

## Methods

### Search strategy and selection criteria

Rapid evidence assessment (REA) methodology was utilised to systematically review the literature for each of the four outcome areas. The REA approach applies rigorous methods for locating, appraising and synthesising the evidence to provide structure, balance and transparency of a practice, but the methodology places restrictions in search criteria due to the breadth of evidence [17]. We searched the following electronic databases with a limited date range of January 2006 to March 2016:

- Cochrane Central Register of Controlled Trials (searched DATE)
- Medline (searched DATE)
- PsycINFO (searched DATE)
- CINAHL (searched DATE)
- PubMed (searched DATE)

Grey literature with a priority focus on reports from government agencies, and quality reports from reputable stakeholders fitting the review scope were also searched. International literature, in English only, that focused on research from HIC, populations and settings was included. Books and book chapters were excluded. An individual search strategy was performed for each outcome area rather than a single over-arching search strategy across all four areas. This gave a better reflection of the flow of studies for each topic at each stage of screening for eligibility. The search was updated in August 2019. The search criteria for each of the topics are included in Additional file 1.

### Eligibility criteria (PICOS format)

#### Participants

Interventions delivered to parent(s) and/or children during the first 2 weeks to 5 years of the child’s life were eligible. The minimum child age was set to 2 weeks to exclude interventions delivered in the first few days after childbirth when the parent/child is potentially still under hospital care. However, studies recruiting in hospitals within 2 weeks of birth were eligible. The limit was set to 5 years to ensure interventions were offered primarily to pre-schoolers, in keeping with the evidence that the early years are central for future development.

Studies focusing exclusively on the following populations were not eligible as they were delivered in completely different health care settings:

- Parent(s) and/or children from low-income countries, populations and settings
- Parent(s) and/or children with a clinical diagnosis of an emotional, behavioural or conduct disorder (e.g. anxiety disorder, ADHD)
- Parent(s) and/or children with specific disabilities/illnesses or comorbidities (e.g. cancer)
- Unique environmental circumstances (e.g. refugee, disaster zone, military families, homeless)

#### Interventions

The current review aimed to identify universal and targeted interventions (selective and indicated) as defined by the United States Institute of Medicine [13]. Studies were excluded if it could not be determined whether the intervention was universal or targeted. Tertiary interventions (e.g. interventions that reduce disability, enhance rehabilitation and prevent relapses and recurrences of the illness) and/or interventions delivered in a tertiary setting were not eligible.

Countries differ in the number of visits/sessions offered as part of universal care, and NICE guidance’s definition of a ‘brief’ intervention extends from ‘a single session or multiple brief sessions’ [18]. In the absence of a universally agreed definition of what is considered a ‘brief’ intervention in child service delivery, we decided to use four sessions as our cut-off. The principal reason for this decision is that, in comparisons of the number of visits recommended in the child health policies of high-income countries of Australia, Canada, USA, Denmark, Finland, Sweden, and Norway [19, 20], the four sessions mandated in the UK is the lowest reported (Health Child Programme, 2009) [21]. Thus, interventions delivered across four sessions could be adapted to even the country with the briefest opportunity to implement (e.g. 5-session interventions automatically preclude adaptation to the UK). In addition, the 4-session definition is used globally for categorising interventions as ‘brief’ for other public health issues of alcohol misuse, smoking, and physical inactivity [22–24]. Interventions that stipulated that parents follow a specific regime outside of the sessions were excluded as (i) intervention fidelity may vary dramatically within participant groups, and (ii) ability to adhere to a schedule may impact parental confidence. These tight inclusion criteria ensured that eligible studies could be adapted for delivery within existing universal child health service structures where only a handful of visits are achievable [25]. No restrictions were placed on the length of time of the intervention sessions.

Interventions delivered by any healthcare practitioner, family member or peer were eligible for inclusion, provided they were deliverable within a UCHS platform. For example, an intervention where clinical psychologists delivered cognitive-behavioural techniques within a tertiary setting would not be eligible but if the same psychologist delivered the same techniques as part of a well-child care program then the intervention could be considered eligible. Telephone-, digital- and internet-based and in-person interventions were all eligible for inclusion if they were delivered in a finite and structured format. Interventions that were not session-based and allowed continual access to support were excluded, for example, online forums where mothers could speak with peers or practitioners at their convenience. Interventions which involved screening but no structured, session-based response for women exceeding screening instrument thresholds were also excluded. Interventions delivered in any setting (e.g. home, community, healthcare) were eligible except for interventions targeting outcomes relating to ‘home learning environment’. Due to the confounding influence of nursery/pre-school/community groups in fostering similar outcomes and the focus of universal services being on the family unit, we stipulated an additional inclusion criterion that infants had to receive the interventions targeting home learning environment within their home. This permitted (i) interventions provided to parents outside of the home but to be delivered to the infant in the home and (ii) interventions delivered directly to the child by intervention provider (e.g. healthcare practitioner). There were no restrictions on the behavioural content used in eligible interventions (e.g. goal setting, self-monitoring, feedback on the behaviour). Lastly, pharmacological interventions were not considered eligible due to their lack of suitably to a universal child services’ platform.

#### Comparison groups

Studies with the following comparison groups were eligible:

1. Usual care pathways, wait-list or no-intervention comparison control groups
2. Assessment-only
3. Leaflet-based information.

Follow up assessments where there was not an equivalent control group comparison would not be reported.

#### Outcomes

To decide the selection of priority areas, an initial ‘long list’ of 24 key topics was generated for consideration by a group of child health clinicians and researchers. The topic list covered indicators that were considered relevant from national frameworks for early childhood health and development [21, 26]. While there was not capacity in the rapid timeframe of the REA to directly consult with members of the public, this was ameliorated by the use of the data from the Child Health Poll, which is a survey of a nationally representative sample of 2000 Australian households with children, and examining the website traffic on the Raising Children Network (an Australian evidence-based parenting website) [27].

A short list of five topics was derived from the ‘long list’ through use of a prioritisation matrix which aimed to score each topic based on (1) prevalence, (2) significant impact to families and communities, and (3) felt to be relevant to current public health and public policy strategic priorities. This was done through a group of experts rating each topic on dimensions of relative prevalence estimates for vulnerable families, relative severity and burden of outcomes, and community interest. This group of experts included paediatricians, researchers, nurses, and the chief advisor on Child & Youth Health to ministry of Health in Australia. The prioritisation matrix informed discussions with the research team to determine which topics should be selected for REA, to ensure that a range of topics were included, particularly given the natural overlap of some topics. The selection process of priority areas is detailed in McLean et al., 2016 [28]. The final topics included for REA were:

- Child social and emotional wellbeing (Child SEWB)
- Infant sleep disorders
- Home learning environment
- Parental mental health

Children with low social and emotional wellbeing (SEWB) are at an increased risk of learning difficulties, academic underachievement, and mental health disorders [29, 30]. Infant sleep duration and quality can have lasting impact on a child’s behavioural, cognitive and physical development without early intervention [31], and increases the likelihood of postnatal depression in mothers from 10 to 45% [32]. The home learning environment is a key determinant of child development. Children who grow up in a poor home learning environment with sub-optimal stimulation have lower levels of educational achievement when they leave school and lower employment levels in adulthood [33, 34]. One in five children has a parent with a mental health disorder [35]. Poor parental mental health is known to increase the risk of social and behavioural problems in childhood and adolescence and increase the child’s risk of developing mental health problems as they get older [36, 37]. Definitions of the priority areas and examples of the outcomes that could be used to measure effects in these areas are presented in Table 1.

**Table 1:** Definition of outcomes

Topic	Definition	Outcomes of interest
**Child social and emotional wellbeing**	Interventions designed to improve, promote and optimise child behavioural outcomes, positive social and/or emotional wellbeing and reduce mental illness in children.	- Externalizing behavioural problems (e.g. oppositional defiance, antisocial behaviour, and aggression) - Internalising behaviour problems (e.g. anxiety, depression) - Infant attachment behaviour
**Infant sleep disorders**	Behavioural and/or education interventions that aim to prevent or improve sleep problems.	- Difficulties falling or staying asleep - Excessive total sleep time - Night waking - Settling problems
**Home learning environment**	Interventions that aim to improve the home learning environment of children by promoting positive intellectual and social development in the child.	- Any relevant cognitive areas (i.e. literacy, pre-literacy, numeracy, pre-numeracy, language and communication, and/or general cognitive functioning). - Frequency of reading, attitudes towards reading - Literacy scores - Language ability - Vocabulary
**Parent mental health**	Interventions that aim to (i) prevent mental illness and promote positive mental health in parents or (ii) improve outcomes of existing mental health problems.	- Rates of diagnoses of mental health disorders (e.g. anxiety, depression) - Self-report on mental health symptom scales (e.g. anxiety, depression)

Interventions may have collected outcome data relating to several areas, but each intervention was categorised as *focusing on* a single outcome area according to the primary outcome or recruited population. The purpose of categorising interventions under a primary outcome area was to see whether uptake may have been influenced by the ‘offer’ of the intervention. For example, if an intervention invited families with infant sleep problems but measured sleep as a primary outcome and parental mental health as a secondary outcome, it was categorised as an infant sleep intervention.

#### Study design

Any study with a comparison group, including randomised controlled trials (RCTs) and quasi-randomised trials were eligible. All other trial designs without an established comparison group were excluded. Systematic reviews were excluded but were searched for relevant studies. Only studies with outcome data collected at least 1 month after intervention delivery were eligible.

### Selection of studies

Data was managed using EPPI-Reviewer 4 software, which is EPPI-Centre’s comprehensive online software tool for research synthesis. Search results for each topic were filtered for duplicates and imported into EPPI-Reviewer 4 software for screening against inclusion/exclusion criteria based on title and abstract. Full-text versions of remaining eligible studies were retrieved and imported to EPPI-Reviewer 4, for full-text screening. Twenty percent of studies were also screened by a second reviewer at the full-text screening stage, to ensure consistency across the project. Consistency of 100% between reviewers was required before studies were accepted for inclusion, and discrepancies were resolved by discussion between reviewers to achieve this. Eligible studies remaining after this final screening were included for review and subject to data extraction.

### Data extraction and analysis

Data from the individual studies were extracted in a consistent format using a form developed for this review. Information extracted for each intervention included details on:

- Approach (universal, selected, indicated)
- Content (what format did the intervention take and what were they targeting)
- Mode of delivery (e.g. telephone, in-person, internet)
- Intensity (number of sessions, length of sessions)
- Provider (who delivered the intervention to participants)
- Effectiveness (outcome data)
- Engagement (recruitment and attrition data)
- Adherence (to what extent did patients complete all the intervention components)

To determine the length of an intervention, the endpoint was defined as the final time participants received intervention content from the intervention provider. Intervention contacts solely for data collection or for following up on participants without new content were not classed as intervention sessions.

Self-report data and observer-reported outcome data (e.g. video-coded behaviour assessment) were extracted. Outcome data not relating to our four outcome areas were not extracted. Data from intention-to-treat analyses were used where reported. Due to variation in the wide range of outcome measures used (both in terms of the outcome areas and/or the instruments used to assess the outcomes), it was not possible to conduct a meta-analysis and results were reported using narrative synthesis of findings.

### Quality appraisal

The National Institute for Clinical Excellence (NICE) quality appraisal checklist for quantitative studies was used to assess study quality (http://www.nice.org.uk/). This checklist considers the appropriateness of the theoretical approach, study design, data collection, trustworthiness, analysis, relevancy of the findings and ethics. Studies received one of the following three potential quality scores:

- ++ (Low risk of bias): All or most of the checklist criteria are fulfilled; where they have not been fulfilled, the conclusions are very unlikely to alter.
- + (Medium risk of bias): Some of the checklist criteria are fulfilled, where they have not been fulfilled, or not adequately described, the conclusions are unlikely to alter.
- (High risk of bias): Few or no checklist criteria are fulfilled and the conclusions are likely or very likely to alter.

Studies were not excluded based on quality but this information was used to consider the conclusions of included studies, and for the interpretation when findings across studies differed. The quality appraisal was used for deciding which interventions may be most suitable for recommending as ‘best bet interventions’. Two trained researchers appraised the quality of each study.

## Results

**Fig. 1:**
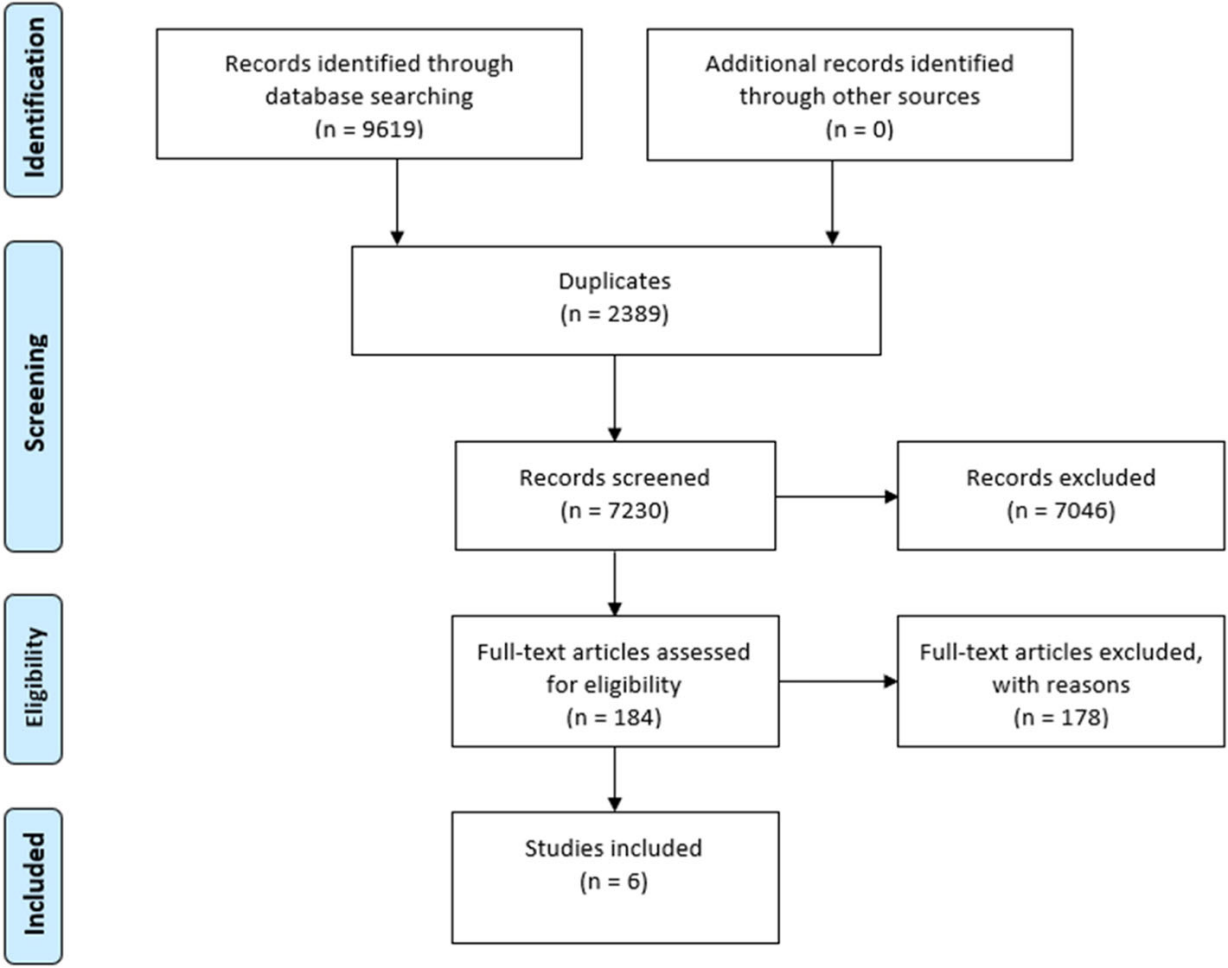
Preferred Reporting Items for Systematic Reviews and Meta-Analyses flow diagram for child social and emotional wellbeing

Figure 1 presents an example PRISMA (Preferred Reporting Items of Systematic Reviews and Meta-Analyses) flow diagram for child SEWB and the other flow diagrams are presented in Additional file 2. Nineteen unique studies were identified across the four searches. Six studies primarily focused on child SEWB [38–43]. Shaw et al. [38] was the only one of these six studies to not also assess parental mental health. Four studies primarily focused on infant sleep outcomes [44–47]: of which, two also assessed parental mental health and child SEWB [44, 45] and one also assessed parental mental health [47]. Five studies focused on home learning environment and reported on no other outcome areas [48–52]. Four studies focused on parental mental health and reported on no other outcome areas [53–57].

A summary of study characteristics for each of the priority area outcomes is presented in Table 2. Although a small number of studies for each priority area, there were some observations: (i) Child SEWB studies were predominantly targeted, low risk of bias, and delivered by healthcare staff, (ii) Home learning environment studies were all universal, without group components and predominantly delivered in healthcare settings, (iii) Infant sleep studies were predominantly single-session and delivered by researchers, and (iv) Parental mental health studies were all universal, and often single -session and delivered by healthcare staff.

**Table 2:** Summary of study characteristics for each priority area

	Child Social & Emotional Wellbeing (***n*** = 6)	Home Learning environment (***n*** = 5)	Infant sleep (***n*** = 4)	Parental mental health (***n*** = 4)
Approach				
Universal	1	5	2	4
Selected/indicated	5	0	2	0
Risk of bias				
High	1	1	1	1
Medium	0	2	1	2
Low	5	2	2	1
Group based component				
Yes	3	0	2	2
No	3	5	2	2
Number of sessions				
1	2	1	3	3
2	1	2	1	0
3	3	1	0	0
4	0	1	0	1
Setting				
Family home	2	1	1	2
Health-related	4	4	3	2
Fields of intervention provider				
Health	5	3	2	3
Social	2	1	0	1
Research	0	0	2	0
Other	2	2	0	0

Table 3 highlights how individual study characteristics are associated with effectiveness whereas Table 4 highlights how indicators of engagement from families is associated with effectiveness. Individual details of the studies are presented in Additional file 3.

1. Child social and emotional wellbeing

**Table 3:**
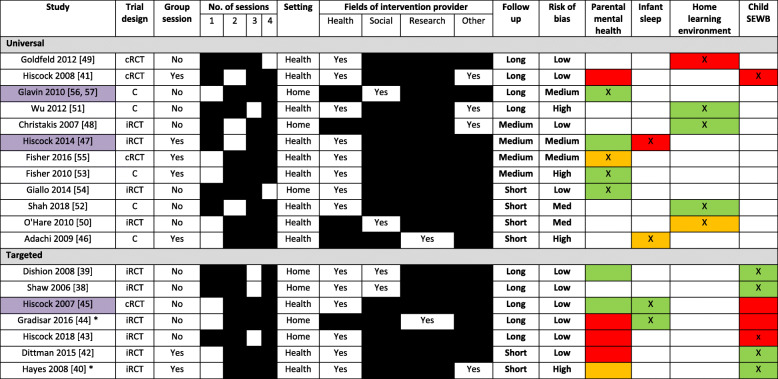
Summary of intervention characteristics and association with effectiveness

*cRCT* Cluster Randomised Controlled Trial, *iRCT* Individually Randomised Controlled Trial, *C* Controlled, *Child SEWB* Child social and emotional wellbeing

Follow up: Short = < 6 months, Medium = 6 months, Long = ≥12 months, Risk of Bias = Assessed by Nice Quality Appraisal Checklist

RAG rating relates to effectiveness: Red = No effect, Amber = Indication of an effect, Green = Significant at the 5% level

* Self-referral to study (e.g. response to advert, contacting triage service)

X = Primary outcome area focused on by intervention

Studies highlighted in purple indicate “Best bet” interventions (significant effect in study with strong methodology and implementable within existing universal child health service)

**Table 4:**
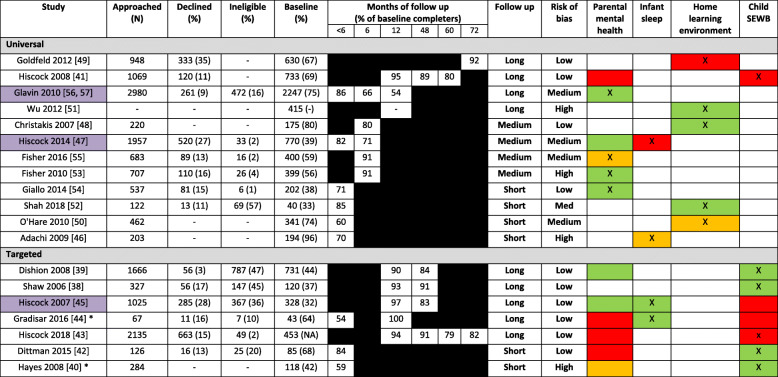
Summary of study engagement and association with effectiveness

*cRCT* Cluster Randomised Controlled Trial, *iRCT* Individually Randomised Controlled Trial, *C* Controlled, *Child SEWB* Child social and emotional wellbeing

Follow up: Short = < 6 months, Medium = 6 months, Long = ≥12 months, Risk of Bias = Assessed by Nice Quality Appraisal Checklist

RAG rating relates to effectiveness: Red = No effect, Amber = Indication of an effect, Green = Significant at the 5% level

* Self-referral to study (e.g. response to advert, contacting triage service)

X = Primary outcome area focused on by intervention

Studies highlighted in purple indicate “Best bet” interventions (significant effect in study with strong methodology and implementable within existing universal child health service)

Of the eight studies that report outcomes relating to child SEWB, six were considered to primarily target child SEWB [38–43] whereas two primarily focused on infant sleep in studies recruiting families that presented with infant sleep problems [44, 45]. Studies examining improvements for child SEWB were mostly well-conducted with 7 of 8 fulfilling all or most of the NICE checklist criteria (Hayes et al. [40] being the exception). The outcome measures selected were comparable across studies (five of the studies used the Child Behaviour Checklist). Despite the robust study designs, the interventions themselves varied considerably in the format they were delivered (e.g. group/individual, home visit/health centre).

From these studies, there is evidence that populations with identified risk factors can benefit from brief interventions that target child SEWB. Specifically, interventions that focused on motivational interviewing and examining family context to identify appropriate needs had benefits 2 years later [38, 39]. Of the two studies primarily targeting improving sleep, Gradisar et al. [44] examined children of comparable ages to those in the other studies whereas Hiscock et al. [45] recruited a younger sample of infants but as their interventions focused on sleep it is not unexpected that child SEWB remained unchanged.

There was little evidence of the effectiveness of universal interventions. Hiscock et al. [41] was both the only (i) universal intervention and (ii) one of two studies targeting child SEWB that did not demonstrate a benefit. A structurally similar group-based intervention also held in maternal child health centres in Melbourne, Australia showed significant improvement in child SEWB [40]. Hiscock’s study [41] received a higher quality appraisal than Hayes’s study [40], but an alternative explanation may be that Hayes et al’s sample had self-referred so may have been more engaged or motivated.

2. Infant sleep

Three of 4 studies tested infant sleep interventions in indicated/selected populations, with Gradisar et al. [44] asking participants to self-refer if their child was experiencing a sleep problem while Hiscock et al’s studies [45, 47] both recruited patients who had been screened for a sleep problem through routine health visits.

The interventions were all essentially single session but differed in the approach taken. There was evidence of effective child behavioural interventions [44, 45] but weak evidence for interventions using parent education alone [46, 47]. Child behavioural interventions may be the ‘best bet’ approach as these interventions were supported by two studies of high methodological quality. Both these studies permitted parents to choose one of two interventions. Interestingly, Gradisar et al. [44] showed that two interventions improved different sleep outcomes (e.g. one reduced number of awakenings whereas the other increased total sleep time).

3. Home learning environment

The five studies measuring outcomes relating to cultivating a positive home learning environment all tested universal interventions that recruited families engaging with routine health visits [48–52]. All five interventions could be delivered within very short timeframes (e.g. waiting rooms, 5-min time slots) or independent of practitioner involvement. However, the studies used different techniques (distribution of books/reading materials/play activities, and literacy promotion programs).

There is currently a paucity of high-quality evidence for brief interventions aiming to improve the home learning environment. Any positive evidence is undermined by methodological issues. Studies reporting positive intervention effects predominantly used non-validated tools devised for the purposes of testing the specific intervention. Goldfeld et al. [49] was the only study not to report any improvement on any outcomes. This study had the highest quality rating and used a variety of validated outcome tools, as such the evidence is more robust and generalisable. Other methodological limitations include follow up time points limited to 6 months or less [48, 50, 52], and no data on the number of participants that were initially approached nor retention rates [51]. Goldfeld et al. [49] had high retention rates at 4-year follow up and as such the findings are more indicative of the long-term impact (or lack) of the intervention.

4. Parent mental health

Twelve studies reported on parental mental health outcomes. Of these, four interventions focus on parent mental health as their primary outcome [53–57], but three interventions primarily focus on infant sleep disorders [44, 45, 47] and five primarily focus on child SEWB [39–43].

Many of the intervention approaches such as individual counselling and psychoeducational programs were delivered in subtly different formats throughout the different trials. Therefore, it is not possible to definitively recommend one implementation method over another. All four interventions targeting parental mental health demonstrated positive results. Interventions targeting parental mental health were all delivered by a nurse and therefore should be adaptable to most universal child health and development programs. All studies apart from Glavin et al. [56, 57] were conducted through existing services in Australia so it is unclear whether they would be applicable within similar contexts. Glavin et al’s counselling intervention was the only intervention modelled on the principle of ‘proportionate universalism’; those from a universal base with increased need received more sessions or referral to additional services.

The group intervention tested in Fisher et al’s studies [53, 55] recruited couples. Further adaption and testing would be required to implement these interventions either with a single parent or a single parent and supportive other. In the study which did not target couples by Giallo et al. [54], the follow up time was limited but findings suggest that self-directed intervention alone is not as beneficial as with telephone support.

The evidence is predominantly negative when the intervention primarily addresses other outcome areas. Among the child SEWB studies, Dishion et al. [39] reported improvements in parental mental health and child SEWB, while Hiscock et al. [40], Dittman et al. [42], and Hiscock et al. [43] demonstrated no improvements in parental mental health. Interestingly, Hayes et al. [40] reported improvements in child SEWB and parental depression, anxiety and stress but the wait-list control group only reported improvements in depression when they received the intervention. Among the sleep studies, intervention groups in both of Hiscock et al’s studies [45, 47] showed greater improvements in depression. Yet, only Hiscock et al. [45] showed an effect on infant sleep outcomes. The inverse was observed by Gradisar et al. [44] as while infant sleep was improved, parental mental health was unaffected.

Whilst this review aimed to assess interventions directed to both maternal and paternal populations, no brief intervention studies were identified that addressed the mental health of fathers. All other studies represented preventative interventions used to mitigate the risk of mothers developing mental illness in the post-partum period.

The evidence suggests that a classic model of services structured on a fixed number of repeated sessions with mothers is not necessary to improve mental health outcomes and that brief interventions can be effective. Consideration should be made to the theoretical underpinnings of interventions to identify the causative links between mental health improvement and intervention components.

### Can these interventions be delivered through a UCHS platform?

Brief interventions should theoretically be acceptable to both families and healthcare practitioners and entail less resources to deliver. From the evidence reviewed we derived data to examine recruitment, adherence and retention rates; providing an indication of the acceptability of these interventions to families to complement the review of effectiveness. Details on indices of engagement are presented in Table 4.

### Uptake

In the 12 studies testing universal interventions, the proportion of participants completing baseline assessments varied across studies from 32.9 to 95.6%, with two studies not providing details on the numbers approached. Eight studies reported the number of participants who explicitly refused to participate. Of these, the refusal rates coming into the studies ranged between 9 to 16% for the studies that focused on parental mental health [53–57], 27% for Hiscock et al’s study targeting infant sleep [47], 11% for Hiscock et al’s study targeting child SEWB [41], and 11 to 35% for studies targeting home learning environment [49, 52]. These low refusal rates suggest that most interventions did appeal to parents. Mental health interventions that could be perceived as stigmatizing were also taken up well by the families.

The six studies that recruited selected/indicated populations either (i) proactively screened participants through routine health visits or directly contacting families by telephone or (ii) advertised the intervention and relied upon participants self-referring. The percentage of participants refusing screening ranged from 3 to 28%. The percentage of participants defined as ineligible after screening ranged from 20 to 47%. It was difficult to determine numbers ineligible and numbers refusing to participate and therefore the extent that the service appeals to patients. Furthermore, there were few details in selected/indicated populations regarding the time and resources for screening against the proportion of patients ultimately eligible.

### Risk factors for non-participation

Twelve of the 19 studies identified in this review stated that sufficient language to complete the assessments was an explicit inclusion criterion. However, any service rolled out on a universal platform would have to explicitly encourage participation from culturally and ethnically diverse populations as many of these populations are at a higher risk of poor parental and child outcomes. Not being a native speaker is a recognised risk factor for not receiving appropriate healthcare resources [58]. Consequently, the interventions may not be generalizable for culturally diverse populations. In addition, several studies highlighted that participation was associated with stress and mood variables [38, 54], indices of social deprivation and socio-economic status [41, 43, 45, 47], levels of education [43, 47, 53, 55], or non-native resident/speaker [47, 54]. This review highlights that socio-economic factors were a barrier to engagement and adherence; even when interventions have been designed to be brief and provided a financial incentive.

### Adherence

While examining uptake and the risk factors for non-participation provide an indication of the initial appeal of the intervention, measures of adherence to the intervention (i.e. completed all aspects) indicate how well interventions engage with and are accepted by families. Even within these brief interventions the number of parents that attended all sessions of the intervention were limited. If brief interventions have been appropriately designed, each session should be designed to impart the maximal amount of information within a limited timeframe. As such, missing a single session may mean that an individual misses vital intervention content that could improve the treatment effect. For example, Fisher et al. [55] found a significantly lower prevalence of mental health diagnoses in those that received the full intervention compared to the group who received usual care, whereas receiving only the partial intervention was not associated with a reduction in prevalence of mental health diagnoses. In addition, the variable rates of attendance for interventions with a limited number of sessions highlights that interventions with a higher number of sessions may have increasing difficulty to retain participants. This is seen even in interventions that recruited participants actively seeking help [40, 44].

### Retention

Encouragingly, retention rates were routinely high across universal studies irrespective of timepoint. Only two studies reported retention rates lower than 70% [50, 56, 57]. Of the targeted interventions, the only two studies with retention rates below 70% were the two studies that recruited through self-referral. Gradisar et al. [44] showed a 54% retention rate at an interim assessment but managed to gain 100% follow up at 12 months. However, Hayes et al. [40] exhibited less than 60% retention at less than 6 months. As this study had high attrition between self-referral and a baseline assessment, it suggests that the parallel triage service may have been a serious confounder.

### Synthesis of evidence: ‘best bet’ interventions

A combination of critical assessment of effectiveness data, indicators of acceptability, and assessments of quality (bias) across all studies was performed to identify potential ‘best bet interventions’ for adoption into UCHS. Studies with a combination of ‘Long’/‘Medium’ follow up, ‘Low’/‘Medium’ risk of bias, and green-coded effectiveness data (Table 2) were critiqued against potential implementation issues to determine whether recommendable in the context of UCHS.

There were two “best bet” interventions identified for potential use in universal services [47, 56, 57]. While Hiscock et al’s [47] child behavioural intervention did not elicit a benefit on sleep outcomes, the intervention was effective at reducing levels of parental depression. As the intervention itself entailed few resources and a single group session we would advocate the use of this intervention for new parents to improve maternal mental health; although there was evidence that those of a lower socio-economic status may be less likely to engage in the intervention. Future research should aim to measure the cost-effectiveness of each part of the program (e.g. DVD, self-help material, group session). We would also recommend Glavin et al’s [56, 57] intervention based on triage for mental health symptoms in all mothers. The intervention was associated with benefits in parent mental health at scale and over a long follow up period. More importantly this intervention was upskilling existing staff to provide additional support as part of universal care making it far more sustainable. The only main limitation is the quasi-experimental approach in which this was tested but as this was a pragmatic trial it is perhaps more reflective of how the intervention would work once implemented in a real-world context. While Christakis et al’s intervention [48] was effective, a fuller understanding of the mechanistic theory underlying the intervention’s benefit is needed along with a longer term follow up that demonstrates the cost-effectiveness of providing the toys used in the intervention.

Of the targeted interventions, we recommend Hiscock et al’s [45] intervention as it effected long term change on both sleep disorders and parental mental health and is feasibly delivered through health centres. In contrast, while Shaw et al. [38], Dishion et al. [39], and Gradisar et al. [44] all demonstrated that their respective interventions were effective at long term change, the feasibility of delivery via existing UCHS has yet to be established as these studies primarily used research staff for delivery. In both Shaw et al. [38] and Dishion et al. [39], participants were financially reimbursed for assessments, which is not feasible for most UCHS; and the same intervention was shown ineffective in a study by Hiscock et al. [43]. In addition, Gradisar et al. [44] had a relatively small sample size that were predominantly in a marriage-like relationship, had education qualifications, and were middle- to high-income earners so has not been tested at scale in families from wider socio-demographic backgrounds.

## Discussion

This restricted evidence assessment on brief interventions to address and promote early childhood health, development, and wellbeing through UCHS suggests that there are several promising effective programs that could be delivered. This is an important finding as early, brief intervention is thought to be a cost-effective strategy [59]. Although recommendations have been based on the potential appropriateness of programs evaluated in a robust trial, it is likely that many of the suggested programs would still require adaptation to be delivered effectively at scale. Interventions for some areas pose a challenge, as there are several similarly designed programs that yielded conflicting results. Nevertheless in order to assist policymakers, service providers, commissioners and/or practitioners in pragmatic (and evidence informed) decision making we have derived some over-arching principles regarding the implementation of brief interventions taking into account evidence of acceptability, effectiveness, and examination of the underlying content and format of interventions. These principles, or ‘lessons learnt’ may assist in the development, implementation and evaluation of brief interventions delivered through UCHS:

1. Brief interventions should be designed to impart the maximal amount of information within an initial session and future sessions should aim to reinforce the key messages rather than provide additional information. These “single session intervention” models would combat variability in adherence and retention rates. The adoption of interventions that were not tested in populations that are potentially the most vulnerable may ultimately widen health inequalities.
2. Brief interventions appear to have high uptake rates and may be more acceptable to potentially stigmatizing areas (e.g. parent mental health). Brief interventions still present considerable barriers for engagement and adherence that may deter the most vulnerable. Future studies should conduct analyses that aim to identify risk factors for non-participation and non-adherence whereas recruitment strategies should be adapted for different populations.
3. Interventions should focus less on the infant themselves but instead see the family as a holistic unit and consider the needs of parents with content having an emphasis on identification of needs, triage and referral.
4. Interventions should (i) be evaluated using validated tools, (ii) present a clear theoretical rationale as to how the intervention components would impact on the outcome measures, and (iii) develop screening criteria for those at-risk of disadvantage. These criteria were noticeably lacking for most home learning environment interventions.
5. Providing a choice of intervention may in itself be an active ingredient to intervention success – recognizing interventions need to be tailored to families’ preference. Services may find it easier to engage parents if they allow parents to identify the issues that they are struggling with and therefore allow them to choose which interventions might help their situation.

In addition to the lessons learnt, there are several more specific findings regarding the content and delivery of interventions and noting that evidence is lacking for each of priority areas. Regarding child SEWB, there is little evidence of the effectiveness of universal interventions and this is an area that requires further research. In contrast, there were no targeted interventions aimed at improving home learning environment and all the universal interventions either showed no improvement or had methodological limitations. Targeted interventions for home learning environment could theoretically be allocated based on screening for recognized risk factors for disadvantages in home learning environment but such interventions need to be developed and tested at scale. In summary, whether interventions are universal or targeted may influence how receptive families are to the intervention and thus be a large determinant of intervention effectiveness.

There is no intervention technique that works across all sleep outcomes but a combination of techniques (e.g. bedtime fading, graduated extinction) may provide the most comprehensively effective approach. Child behavioural interventions may be the ‘best bet’ approach for infant sleep problems rather than bedtime routine interventions or parent information alone. Positive findings were found for different behavioural techniques (e.g. bedtime fading, graduated extinction). As techniques were not mutually exclusive, a combination or choice may maximize on the number of infant sleep areas that are amenable to change. Alternatively, it may be best to tailor the sleep management strategy to what is the most concerning for families. The interventions identified as effective are all suitable for younger children but is unclear whether they would be equally effective in pre-school children as there was a paucity of evidence relating to the effectiveness of any sleep intervention in preschooler children (age 3–5).

For the other outcome areas there was uncertainty on *how* the intervention may exert an effect. Home learning environment interventions may have additional benefits by guiding parents in how to interact with their child better but parental mental health and child SEWB outcomes were not reported. Similarly in Fisher et al’s studies [53, 55], the parental mental health intervention recruited couples and so the intervention benefits may have emerged through fostering better understanding of parenting behaviors between partners, rather than teaching strategies that explicitly address mental health. These studies suggest that the relationship between child and parent outcomes are complex, and more consideration of mechanism of action is required.

Regarding the structure of interventions, it could be argued that across outcomes areas, in a brief intervention that is not dependent on repeated contact with a provider for monitoring progress, an initial session may provide sufficient intervention content to elicit an effect and follow up sessions merely provide reinforcement of key messages.

### Implementation challenges

Workforce capacity remains a major consideration for the implementation of these brief interventions within the context of universal child and family services. A consistent finding across topic areas and individual studies was the relative lack of detail provided regarding workforce capacity issues. However, there is a great deal of promise with many of the recommended programs being delivered by existing universal service nurses or by other existing community practitioners. Training of existing staff is beneficial in that it is building upon existing structures, such as Glavin et al. [56, 57] improving child health nurses’ abilities to monitor and treat mental illness. Studies which required trained research staff or highly trained, specialized professionals to deliver the interventions are less generalisable. Embedding the same level of intervention within existing work structures may not be feasible in the long-term. The costs associated with training or hiring appropriately qualified staff would require further consideration in terms of financial viability as well as operational and logistical issues. Even within our ‘best bet’ interventions, “upskilling” of existing UCHS staff is required and the foundational training and qualifications of UCHS workers differ by country. Thus, while our ‘best bet’ intervention may help signpost commissioners and practitioners to identify relevant interventions, they should also consider the extent that the intervention can be adapted to their specific setting, and that appropriate feasibility evaluation is conducted to examine whether comparable effectiveness is shown once implemented.

Proportionate universalism is designed to provide additional support to families at greatest need. There is some debate about the best way to identify those who require additional assistance. One approach has been to use predefined general risk factors that identify vulnerability. However, it has also been argued that it should be “need” rather than risk factors alone that identify families, with the benefits of efficiency (better targeting) and parental acceptance of the services. This latter approach would then require tools used to identify concerns and problems. The generalisability and applicability of services focusing on risk factor indicators versus identification of need is an important distinction that requires further discussion. Regardless, it is promising that there are several studies that report positive outcomes for vulnerable groups. The challenge will be to determine if programs are able to be adapted for wider demographics if necessary. Good examples of this are the Hiscock et al. [45] sleep trial that was specifically designed to be delivered equally to families of low, middle and high socio-economic status and the Glavin et al. [56, 57] trial that triaged according to parent mental health screening. Following the issue of identification/triage, for early intervention to be successful there must be tools that can accurately identify “issues” for remediation. While, an evaluation of the measurement tools was beyond the scope of this review, it is a key element that should be considered in the broader context of implementation. This point is relevant for all the outcome areas covered in this review. Further effective screening tools may be required for identifying parents with mental health problems and parenting issues, and for identifying children with sleep and social and emotional issues.

### Limitations

The review covered four areas for which there was a large body of research, and so a REA was conducted with a tight inclusion criterion to limit the breadth of evidence. Part of the selection of topic areas involved a survey of only Australian participants for cross-validation from the public but the initial sourcing of topic areas from international policy documents, inclusion of international participants in the prioritisation exercise and ultimately the final consensus from an international group ensured these topic areas were of international importance. Consequently, a few potentially relevant interventions may have been missed. However, rather than provide an exhaustive presentation of all brief interventions and advocate a specific program, this REA gives an overview of potentially usable interventions and provides principles of what could be adapted and where further research is required in the field.

The focus of this REA was on interventions that provide generalised support to common problems for primarily preventative purposes, rather than on interventions aiming to treat patients with a clinically detectable problem. As such the interventions should be brief and not be considered high intensity. There is no global definition of what constitutes ‘brief’ in child health services and there is variation across HICs in the number of universal visits available through which to deliver interventions. We used a four-session cut-off as this is the maximum number of sessions that the HIC with the lowest number of sessions (UK) has available to deliver an intervention. Though not universal, we hope this definition prevents excluding any interventions that could be implemented across HICs and provides critique of a suite of interventions that commissioners/practitioners may choose to adopt depending on their specific settings*.* However, our definition of ‘brief’ as four sessions could still be over-inclusive. The Making Every Contact Count (MECC) approach emphasises using daily interactions to support people making positive changes to their physical and mental health and wellbeing. It is centred on ‘brief interventions’ (defined as oral discussion, negotiation or encouragement, which may involve referral for further interventions or more intensive support) and ‘very brief intervention’ (defined as taking from 30 seconds to a couple of minutes to enables the delivery of information, or signposting to further help) [18, 60]. Adherence to these definitions would have severely limited the number of studies that could be feasibly implemented within existing service provision. Furthermore, the ‘intervention’ in the circumstances are primarily focused on the referral and signposting, rather than active intervention. In contrast, NICE guidance defines ‘extended brief interventions’ as involving ‘a single session or multiple brief sessions’ which is open-ended. Half of the studies included in this review were delivered in a single session, and many were structured to use subsequent sessions primarily for reinforcing information from the initial session, and as such we feel we provide an overview of existing interventions that meet the MECC approach criteria but also allow critical evaluation of slightly longer interventions that commissioners may be able to implement within their existing service provision. It is important to consider that the longer interventions run for, practitioners may utilise more extensive behaviour change techniques other than information-giving, such as action planning, demonstration of the behaviour and feedback on the behaviour/goal setting. Policy guidance needs to work further on standardised definitions on what constitutes ‘brief’ interventions so that commissioners have more insight into what is typical service provision across the sector; and this may be better defined by intervention content rather than length.

It is beyond the scope of this REA to give a comprehensive evaluation of the cost implications for implementation, however there were some examples where the interventions appear to be more cost effective than control or usual care conditions. For example, the provision of individual sleep management plan – “Controlled Crying” or “Camping Out” trialled by Hiscock et al. [45] and several telephone interventions could prove to be cost-effective. The financial investment required for each of the interventions requires further investigation; in particular, the large-scale universal approaches that entailed distribution of physical materials and resources to families (e.g. books, toys, workbooks). Interventions without these physical materials can be assumed to incur less cost.

A very important consideration in the implementation of any of the recommended interventions is the sustainability, or ‘sleeper effects’ of any positive outcomes. Whilst any improvement in the important issues investigated is a positive and worthy outcome, given the significant amount of resources associated with program implementation, the programs with the most sustained benefits should be given higher priority. Few studies measured long term outcomes. However, it was encouraging that benefits could be seen with these brief interventions as it could be presumed that more disadvantaged populations may need more intensive intervention programmes than brief interventions can offer.

## Conclusions

This REA identified evidence of several brief interventions that were effective in helping families manage and promote child SEWB, infant sleep, the home learning environment, and parental mental health. Of these, we present three interventions that we recommend be evaluated at scale from UCHS platforms: (1) a universal child behavioural intervention which did not affect its primary outcome of infant sleep but significantly improve parental mental health, (2) a universal screening programme which significantly improved maternal mental health, and (3) a targeted child behavioural intervention which significantly improved parent-reported infant sleep problems and parental mental health. In addition, a set of “lessons learnt” suggest how brief interventions targeting these outcome areas should be structured, delivered, and tested. The implementation of appropriate and brief evidence-based interventions in UCHS could lead to the development of a more responsive and equitable service that better identifies and meets the needs of children and families to promote early childhood development.

## Supplementary information

**Additional file 1.** Search strategies (Word document).

**Additional file 2.** Prisma flow diagrams (Word document).

**Additional file 3.** Table of individual studies (Word document).

## Data Availability

The datasets used and/or analysed during the current study are available from the corresponding author on reasonable request.

## Abbreviations

UCHS: Universal child health services
Child SEWB: Child Social and Emotional Wellbeing
