# Supplementary material for: Brief evidence-based interventions for universal child health services: a restricted evidence assessment of the literature
Source: BMC Public Health. 2020 Jun 24;20:993. doi: 10.1186/s12889-020-09104-7 (PMC7315474; doi:10.1186/s12889-020-09104-7)
Supplement: Supplementary file 1 — Additional file 1. Search strategies (Word document). [file 12889_2020_9104_MOESM1_ESM.docx]

**Additional file 1. Search strategies**

Child social and emotional wellbeing search strategy

The following is an example of the search strategy conducted in Medline

| **Step** | **Search terms** | **No. of records** |
| --- | --- | --- |
| S1 | Exp Child/ | 870289 |
| S2 | Exp Infant/ | 490456 |
| S3 | (neonat* or infan* or pre-schooler* or pre-schooler* or under-five* or p?ediatric*).tw,kf,hw. | 716978 |
| S4 | 1 or 2 or 3 | 1235825 |
| S5 | Exp child health services/ or program*.tw,kf,hw. or intervention*.tw,kf,hw. or parenting.mp. | 1009223 |
| S6 | Bonding or attachment.mp. or reactive attachment disorder/ | 118917 |
| S7 | *anxiety, separation/ or *panic disorder/ or *child behavior disorders/ | 10693 |
| S8 | externalizing problem*.mp. | 1364 |
| S9 | internalizing problem*.mp | 1120 |
| S10 | *Conduct disorder/ | 1638 |
| S11 | *Adjustment disorders/ | 690 |
| S12 | Social anxiety.mp. | 3351 |
| S13 | *Depression/ | 38370 |
| S14 | Mental disorder.mp. | 4362 |
| S15 | Emotional wellbeing.mp. | 280 |
| S16 | 6 or 7 or 8 or 9 or 10 or 11 or 12 or 13 or 14 or 15 | 59690 |
| S17 | 4 and 5 and 16 | 3386 |
| S18 | Developing countries/ | 73972 |
| S19 | (Austere or (limited adj2 resource*) or (low adj2 resource*) or transitioning econom* or emerging countr* or developing countr* or (("low income" or "middle income" or "low to middle income") and countr*) or "third world" or (underdeveloped adj countr*) or (under adj developed adj countr*) or LMIC).mp. | 77824 |
| S20 | Exp africa/ | 135947 |
| S21 | Americas/ or exp caribbean region/ or exp central america/ or latin america/ or mexico/ or exp south america/ | 131234 |
| S22 | Europe/ or exp europe, eastern/ or exp transcaucasia/ | 145729 |
| S23 | Antarctic regions/ or exp atlantic islands/ or exp indian ocean islands/ or exp pacific islands/ | 41855 |
| S24 | New Guinea/ | 309 |
| S25 | Asia/ or exp asia, central/ or asia, southeastern/ or borneo/ or cambodia/ or east timor/ or indonesia/ or laos/ or malaysia/ or mekong valley/ or myanmar/ or philippines/ or thailand/ or vietnam/ or asia, western/ or bangladesh/ or bhutan/ or india/ or middle east/ or afghanistan/ or iran/ or iraq/ or jordan/ or lebanon/ or oman/ or saudi arabia/ or syria/ or turkey/ or yemen/ or nepal/ or pakistan/ or sri lanka/ or far east/ or china/ or tibet/ or exp korea/ or mongolia/ | 311833 |
| S26 | Africa or americas or caribbean or "central america" or "latin america" or "south america" or "eastern europe" or Transcaucasia or antarctic or (atlantic adj island*) or (indian adj ocean adj island*) or (pacific adj island*) or polynesia or "central asia" or (southeast* adj asia) or (south adj east* adj asia) or borneo or mekong or "western asia" or "middle east" or "far east").mp. | 129096 |
| S27 | (Afghanistan or Albania or Algeria or Angola or Antigua or Argentina or Armenia or Azerbaijan or Bangladesh or Barbados or Barbuda or Belarus or Belize or Benin or Bhutan or Bolivia or Bosnia or Botswana or Brazil or Bulgaria or "Burkina Faso" or Burma or Burundi or Cambodia or Cameroon or "Cape Verde" or "Cabo Verde" or "Central African Republic" or Chad or Chile or China or Colombia or Comoros or Congo or Kongo or (Cook adj Island*) or "Costa Rica" or "Cote D'ivoire" or Croatia or Cuba or "Czech Republic" or Czechoslovakia or Djibouti or Dominica or Dominican or "East Timor" or Ecuador or Egypt or "El Salvador" or "Equatorial Guinea" or Eritrea or Estonia or Ethiopia or Fiji or Futuna or Gabon or Gambia or Gaza or Georgia or Ghana or Grenada or Guatemala or Guinea or "Guinea Bissau" or Guyana or Haiti or Herzeg* or Honduras or Hungary or India or Indonesia or Iran or Iraq or "Ivory Coast" or Jamaica or Jordan or Kazakhstan or Kenya or Kiribati or Korea or Kosovo or "Kyrgyz Republic" or Kyrgyzstan or Laos or (Lao adj People* adj Democratic adj Republic) or "Lao PDR" or Latvia or Lebanon or Lesotho or Liberia or Libya or Lithuania or Macedonia or Madagascar or Malawi or Malaysia or Maldives or Mali or (Marshall adj Island*) or Mauritania or Mauritius or Mexico or Micronesia or Moldova or Mongolia or Montserrat or Montenegro or Morocco or Mozambique or Myanmar or Namibia or Nauru or Nepal or "New Guinea" or Nicaragua or Niue or Niger or Nigeria or Oman or Pakistan or Palau or Panama or "Papua New Guinea" or Paraguay or Peru or Philippines or Poland or Yemen or Romania or Russia or Rwanda or "Saint Kitts Nevis" or "St Kitts Nevis" or "Saint Vincent Grenadines" or Samoa or "St Vincent Grenadines" or "Saint Lucia" or "St Lucia" or "Saint Helena" or "St Helena" or "Sao Tome Principe" or "Saudi Arabia" or Senegal or Serbia or Seychelles or "Sierra Leone" or Slovak or "South Africa" or Solomon Island* or Somalia or "Sri Lanka" or Sudan or Suriname or Swaziland or Syria or Tajikistan or Tanzania or Thailand or Tibet or "Timor-Leste" or Togo or Tokelau or Tonga or Trinidad or Tobago or Tunisia or Turkey or Turkmenistan or Tuvalu or Uganda or Ukraine or Uruguay or Uzbekistan or Vanuatu or Venezuela or Vietnam or "Wallis Futuna" or "West Bank" or Yemen or Zaire or Zambia or Zimbabwe).mp. | 770632 |
| S28 | 18 or 19 or 20 or 21 or 22 or 23 or 24 or 25 or 26 or 27 | 949838 |
| S29 | Limit 17 to (English language and yr=”2006-current”) | 2281 |
| S30 | 29 not 28 | 2060 |
| S31 | Limit 30 to (“all infant (birth to 23 months)” or “newborn infant (birth to one month)” or “infant (1 to 23 months)” or “preschool child (2 to 5 years)”) | 1484 |

Infant sleep search strategy

The following is an example of the search strategy conducted in Medline

| **Step** | **Search terms** | **No. of records** |
| --- | --- | --- |
| S1 | Exp Child/ | 870289 |
| S2 | Exp Infant/ | 490456 |
| S3 | (neonat* or infan* or pre-schooler* or pre-schooler* or under-five* or p?ediatric*).tw,kf,hw. | 716978 |
| S4 | 1 or 2 or 3 | 1235825 |
| S5 | Exp Parent/ | 60366 |
| S6 | Exp Parent child relations/ | 26314 |
| S7 | (mother* or father*).tw,kf,hw. | 125154 |
| S8 | 5 or 6 or 7 | 162677 |
| S9 | Exp Child health services/ | 11835 |
| S10 | (intervention* or program*).tw,kf,hw. | 993098 |
| S11 | 9 or 10 | 999120 |
| S12 | (Sleep/ph or sleep wake disorder/px or sleep wake disorder/th or sleep wake disorder/pc or sleep duration.tw,kf,hw. or exp crying/) | 19183 |
| S13 | Developing countries/ | 73972 |
| S14 | (Austere or (limited adj2 resource*) or (low adj2 resource*) or transitioning econom* or emerging countr* or developing countr* or (("low income" or "middle income" or "low to middle income") and countr*) or "third world" or (underdeveloped adj countr*) or (under adj developed adj countr*) or LMIC).mp. | 77824 |
| S15 | Exp africa/ | 135947 |
| S16 | Americas/ or exp caribbean region/ or exp central america/ or latin america/ or mexico/ or exp south america/ | 131234 |
| S17 | Europe/ or exp europe, eastern/ or exp transcaucasia/ | 145729 |
| S18 | Antarctic regions/ or exp atlantic islands/ or exp indian ocean islands/ or exp pacific islands/ | 41855 |
| S19 | New Guinea/ | 309 |
| S20 | Asia/ or exp asia, central/ or asia, southeastern/ or borneo/ or cambodia/ or east timor/ or indonesia/ or laos/ or malaysia/ or mekong valley/ or myanmar/ or philippines/ or thailand/ or vietnam/ or asia, western/ or bangladesh/ or bhutan/ or india/ or middle east/ or afghanistan/ or iran/ or iraq/ or jordan/ or lebanon/ or oman/ or saudi arabia/ or syria/ or turkey/ or yemen/ or nepal/ or pakistan/ or sri lanka/ or far east/ or china/ or tibet/ or exp korea/ or mongolia/ | 311833 |
| S21 | Africa or americas or caribbean or "central america" or "latin america" or "south america" or "eastern europe" or Transcaucasia or antarctic or (atlantic adj island*) or (indian adj ocean adj island*) or (pacific adj island*) or polynesia or "central asia" or (southeast* adj asia) or (south adj east* adj asia) or borneo or mekong or "western asia" or "middle east" or "far east").mp. | 129096 |
| S22 | (Afghanistan or Albania or Algeria or Angola or Antigua or Argentina or Armenia or Azerbaijan or Bangladesh or Barbados or Barbuda or Belarus or Belize or Benin or Bhutan or Bolivia or Bosnia or Botswana or Brazil or Bulgaria or "Burkina Faso" or Burma or Burundi or Cambodia or Cameroon or "Cape Verde" or "Cabo Verde" or "Central African Republic" or Chad or Chile or China or Colombia or Comoros or Congo or Kongo or (Cook adj Island*) or "Costa Rica" or "Cote D'ivoire" or Croatia or Cuba or "Czech Republic" or Czechoslovakia or Djibouti or Dominica or Dominican or "East Timor" or Ecuador or Egypt or "El Salvador" or "Equatorial Guinea" or Eritrea or Estonia or Ethiopia or Fiji or Futuna or Gabon or Gambia or Gaza or Georgia or Ghana or Grenada or Guatemala or Guinea or "Guinea Bissau" or Guyana or Haiti or Herzeg* or Honduras or Hungary or India or Indonesia or Iran or Iraq or "Ivory Coast" or Jamaica or Jordan or Kazakhstan or Kenya or Kiribati or Korea or Kosovo or "Kyrgyz Republic" or Kyrgyzstan or Laos or (Lao adj People* adj Democratic adj Republic) or "Lao PDR" or Latvia or Lebanon or Lesotho or Liberia or Libya or Lithuania or Macedonia or Madagascar or Malawi or Malaysia or Maldives or Mali or (Marshall adj Island*) or Mauritania or Mauritius or Mexico or Micronesia or Moldova or Mongolia or Montserrat or Montenegro or Morocco or Mozambique or Myanmar or Namibia or Nauru or Nepal or "New Guinea" or Nicaragua or Niue or Niger or Nigeria or Oman or Pakistan or Palau or Panama or "Papua New Guinea" or Paraguay or Peru or Philippines or Poland or Yemen or Romania or Russia or Rwanda or "Saint Kitts Nevis" or "St Kitts Nevis" or "Saint Vincent Grenadines" or Samoa or "St Vincent Grenadines" or "Saint Lucia" or "St Lucia" or "Saint Helena" or "St Helena" or "Sao Tome Principe" or "Saudi Arabia" or Senegal or Serbia or Seychelles or "Sierra Leone" or Slovak or "South Africa" or Solomon Island* or Somalia or "Sri Lanka" or Sudan or Suriname or Swaziland or Syria or Tajikistan or Tanzania or Thailand or Tibet or "Timor-Leste" or Togo or Tokelau or Tonga or Trinidad or Tobago or Tunisia or Turkey or Turkmenistan or Tuvalu or Uganda or Ukraine or Uruguay or Uzbekistan or Vanuatu or Venezuela or Vietnam or "Wallis Futuna" or "West Bank" or Yemen or Zaire or Zambia or Zimbabwe).mp. | 770632 |
| S24 | 13 or 14 or 15 or 16 or 17 or 18 or 19 or 20 or 21 or 22 or 23 | 949838 |
| S25 | ((4 and 8) and 4) and 11 and 12 | 670 |
| S26 | Limit 25 to (English language and year “2006-current”) | 473 |
| S27 | 26 nor 24 | 429 |

Home learning environment search strategy

The following is an example of the search strategy conducted in Medline

| **Step** | **Search terms** | **No. of records** |
| --- | --- | --- |
| S1 | Exp Child/ | 870289 |
| S2 | Exp Infant/ | 490456 |
| S3 | (neonat* or infan* or pre-schooler* or pre-schooler* or under-five* or p?ediatric*).tw,kf,hw. | 716978 |
| S4 | 1 or 2 or 3 | 1235825 |
| S5 | Exp child health services/ or program*.tw,kf,hw. or intervention*.tw,kf,hw. or parenting.mp. | 1009223 |
| S6 | Home learning.mp. or *learning/ or cognitive training.mp. or (play and playthings).mp. or *problem solving/ or *literacy/ or *learning/ or *educational status/ or *cognition/ or academic achievement.mp. or *early intervention, education/ or *language development/ | 74120 |
| S7 | 4 and 5 and 6 | 3049 |
| S8 | Limit 7 to (English language and year “2006-current”) | 2166 |
| S9 | Developing countries/ | 73972 |
| S10 | (Austere or (limited adj2 resource*) or (low adj2 resource*) or transitioning econom* or emerging countr* or developing countr* or (("low income" or "middle income" or "low to middle income") and countr*) or "third world" or (underdeveloped adj countr*) or (under adj developed adj countr*) or LMIC).mp. | 77824 |
| S11 | Exp africa/ | 135947 |
| S12 | Americas/ or exp caribbean region/ or exp central america/ or latin america/ or mexico/ or exp south america/ | 131234 |
| S13 | Europe/ or exp europe, eastern/ or exp transcaucasia/ | 145729 |
| S14 | Antarctic regions/ or exp atlantic islands/ or exp indian ocean islands/ or exp pacific islands/ | 41855 |
| S15 | New Guinea/ | 309 |
| S16 | Asia/ or exp asia, central/ or asia, southeastern/ or borneo/ or cambodia/ or east timor/ or indonesia/ or laos/ or malaysia/ or mekong valley/ or myanmar/ or philippines/ or thailand/ or vietnam/ or asia, western/ or bangladesh/ or bhutan/ or india/ or middle east/ or afghanistan/ or iran/ or iraq/ or jordan/ or lebanon/ or oman/ or saudi arabia/ or syria/ or turkey/ or yemen/ or nepal/ or pakistan/ or sri lanka/ or far east/ or china/ or tibet/ or exp korea/ or mongolia/ | 311833 |
| S17 | Africa or americas or caribbean or "central america" or "latin america" or "south america" or "eastern europe" or Transcaucasia or antarctic or (atlantic adj island*) or (indian adj ocean adj island*) or (pacific adj island*) or polynesia or "central asia" or (southeast* adj asia) or (south adj east* adj asia) or borneo or mekong or "western asia" or "middle east" or "far east").mp. | 129096 |
| S18 | (Afghanistan or Albania or Algeria or Angola or Antigua or Argentina or Armenia or Azerbaijan or Bangladesh or Barbados or Barbuda or Belarus or Belize or Benin or Bhutan or Bolivia or Bosnia or Botswana or Brazil or Bulgaria or "Burkina Faso" or Burma or Burundi or Cambodia or Cameroon or "Cape Verde" or "Cabo Verde" or "Central African Republic" or Chad or Chile or China or Colombia or Comoros or Congo or Kongo or (Cook adj Island*) or "Costa Rica" or "Cote D'ivoire" or Croatia or Cuba or "Czech Republic" or Czechoslovakia or Djibouti or Dominica or Dominican or "East Timor" or Ecuador or Egypt or "El Salvador" or "Equatorial Guinea" or Eritrea or Estonia or Ethiopia or Fiji or Futuna or Gabon or Gambia or Gaza or Georgia or Ghana or Grenada or Guatemala or Guinea or "Guinea Bissau" or Guyana or Haiti or Herzeg* or Honduras or Hungary or India or Indonesia or Iran or Iraq or "Ivory Coast" or Jamaica or Jordan or Kazakhstan or Kenya or Kiribati or Korea or Kosovo or "Kyrgyz Republic" or Kyrgyzstan or Laos or (Lao adj People* adj Democratic adj Republic) or "Lao PDR" or Latvia or Lebanon or Lesotho or Liberia or Libya or Lithuania or Macedonia or Madagascar or Malawi or Malaysia or Maldives or Mali or (Marshall adj Island*) or Mauritania or Mauritius or Mexico or Micronesia or Moldova or Mongolia or Montserrat or Montenegro or Morocco or Mozambique or Myanmar or Namibia or Nauru or Nepal or "New Guinea" or Nicaragua or Niue or Niger or Nigeria or Oman or Pakistan or Palau or Panama or "Papua New Guinea" or Paraguay or Peru or Philippines or Poland or Yemen or Romania or Russia or Rwanda or "Saint Kitts Nevis" or "St Kitts Nevis" or "Saint Vincent Grenadines" or Samoa or "St Vincent Grenadines" or "Saint Lucia" or "St Lucia" or "Saint Helena" or "St Helena" or "Sao Tome Principe" or "Saudi Arabia" or Senegal or Serbia or Seychelles or "Sierra Leone" or Slovak or "South Africa" or Solomon Island* or Somalia or "Sri Lanka" or Sudan or Suriname or Swaziland or Syria or Tajikistan or Tanzania or Thailand or Tibet or "Timor-Leste" or Togo or Tokelau or Tonga or Trinidad or Tobago or Tunisia or Turkey or Turkmenistan or Tuvalu or Uganda or Ukraine or Uruguay or Uzbekistan or Vanuatu or Venezuela or Vietnam or "Wallis Futuna" or "West Bank" or Yemen or Zaire or Zambia or Zimbabwe).mp. | 770632 |
| S19 | 9 or 10 or 11 or 12 or 13 or 14 or 15 or 16 or 17 or 18 | 949838 |
| S20 | 8 not 19 | 1918 |

Parent mental health search strategy

The following is an example of the search strategy conducted in Medline

| **Step** | **Search terms** | **No. of records** |
| --- | --- | --- |
| S1 | Exp Child/ | 870289 |
| S2 | Exp Infant/ | 490456 |
| S3 | (neonat* or infan* or pre-schooler* or pre-schooler* or under-five* or p?ediatric*).tw,kf,hw. | 716978 |
| S4 | 1 or 2 or 3 | 1235825 |
| S5 | Exp Parent/ | 60366 |
| S6 | Exp Parent child relations/ | 26314 |
| S7 | (mother* or father*).tw,kf,hw. | 125154 |
| S8 | 5 or 6 or 7 | 162677 |
| S9 | Exp Child health services/ | 11835 |
| S10 | (intervention* or program*).tw,kf,hw. | 993098 |
| S11 | 9 or 10 | 999120 |
| S12 | *Anxiety disorders/ | 12450 |
| S13 | *Depression, Postpartum/ | 3379 |
| S14 | *Depressive disorder/ | 27648 |
| S15 | *Stress, psychological/ | 42697 |
| S16 | Parenting, psychology*.tw,kf,hw. | 11 |
| S17 | Emotional wellbeing.tw,kf,hw. | 280 |
| S18 | *Resilience, psychological/ | 1648 |
| S19 | *Depression/ | 38370 |
| S20 | *Mental disorders/ | 53777 |
| S21 | 12 or 13 or 14 or 15 or 16 or 17 or 18 or 19 or 20 | 168684 |
| S22 | ((4 and 8) and 4) and 11 and 23 | 1928 |
| S23 | Developing countries/ | 73972 |
| S24 | (Austere or (limited adj2 resource*) or (low adj2 resource*) or transitioning econom* or emerging countr* or developing countr* or (("low income" or "middle income" or "low to middle income") and countr*) or "third world" or (underdeveloped adj countr*) or (under adj developed adj countr*) or LMIC).mp. | 77824 |
| S25 | Exp africa/ | 135947 |
| S26 | Americas/ or exp caribbean region/ or exp central america/ or latin america/ or mexico/ or exp south america/ | 131234 |
| S27 | Europe/ or exp europe, eastern/ or exp transcaucasia/ | 145729 |
| S28 | Antarctic regions/ or exp atlantic islands/ or exp indian ocean islands/ or exp pacific islands/ | 41855 |
| S29 | New Guinea/ | 309 |
| S30 | Asia/ or exp asia, central/ or asia, southeastern/ or borneo/ or cambodia/ or east timor/ or indonesia/ or laos/ or malaysia/ or mekong valley/ or myanmar/ or philippines/ or thailand/ or vietnam/ or asia, western/ or bangladesh/ or bhutan/ or india/ or middle east/ or afghanistan/ or iran/ or iraq/ or jordan/ or lebanon/ or oman/ or saudi arabia/ or syria/ or turkey/ or yemen/ or nepal/ or pakistan/ or sri lanka/ or far east/ or china/ or tibet/ or exp korea/ or mongolia/ | 311833 |
| S31 | Africa or americas or caribbean or "central america" or "latin america" or "south america" or "eastern europe" or Transcaucasia or antarctic or (atlantic adj island*) or (indian adj ocean adj island*) or (pacific adj island*) or polynesia or "central asia" or (southeast* adj asia) or (south adj east* adj asia) or borneo or mekong or "western asia" or "middle east" or "far east").mp. | 129096 |
| S32 | (Afghanistan or Albania or Algeria or Angola or Antigua or Argentina or Armenia or Azerbaijan or Bangladesh or Barbados or Barbuda or Belarus or Belize or Benin or Bhutan or Bolivia or Bosnia or Botswana or Brazil or Bulgaria or "Burkina Faso" or Burma or Burundi or Cambodia or Cameroon or "Cape Verde" or "Cabo Verde" or "Central African Republic" or Chad or Chile or China or Colombia or Comoros or Congo or Kongo or (Cook adj Island*) or "Costa Rica" or "Cote D'ivoire" or Croatia or Cuba or "Czech Republic" or Czechoslovakia or Djibouti or Dominica or Dominican or "East Timor" or Ecuador or Egypt or "El Salvador" or "Equatorial Guinea" or Eritrea or Estonia or Ethiopia or Fiji or Futuna or Gabon or Gambia or Gaza or Georgia or Ghana or Grenada or Guatemala or Guinea or "Guinea Bissau" or Guyana or Haiti or Herzeg* or Honduras or Hungary or India or Indonesia or Iran or Iraq or "Ivory Coast" or Jamaica or Jordan or Kazakhstan or Kenya or Kiribati or Korea or Kosovo or "Kyrgyz Republic" or Kyrgyzstan or Laos or (Lao adj People* adj Democratic adj Republic) or "Lao PDR" or Latvia or Lebanon or Lesotho or Liberia or Libya or Lithuania or Macedonia or Madagascar or Malawi or Malaysia or Maldives or Mali or (Marshall adj Island*) or Mauritania or Mauritius or Mexico or Micronesia or Moldova or Mongolia or Montserrat or Montenegro or Morocco or Mozambique or Myanmar or Namibia or Nauru or Nepal or "New Guinea" or Nicaragua or Niue or Niger or Nigeria or Oman or Pakistan or Palau or Panama or "Papua New Guinea" or Paraguay or Peru or Philippines or Poland or Yemen or Romania or Russia or Rwanda or "Saint Kitts Nevis" or "St Kitts Nevis" or "Saint Vincent Grenadines" or Samoa or "St Vincent Grenadines" or "Saint Lucia" or "St Lucia" or "Saint Helena" or "St Helena" or "Sao Tome Principe" or "Saudi Arabia" or Senegal or Serbia or Seychelles or "Sierra Leone" or Slovak or "South Africa" or Solomon Island* or Somalia or "Sri Lanka" or Sudan or Suriname or Swaziland or Syria or Tajikistan or Tanzania or Thailand or Tibet or "Timor-Leste" or Togo or Tokelau or Tonga or Trinidad or Tobago or Tunisia or Turkey or Turkmenistan or Tuvalu or Uganda or Ukraine or Uruguay or Uzbekistan or Vanuatu or Venezuela or Vietnam or "Wallis Futuna" or "West Bank" or Yemen or Zaire or Zambia or Zimbabwe).mp. | 770632 |
| S33 | 23 or 24 or 25 or 26 or 27 or 28 or 29 or 30 or 31 or 32 | 949838 |
| S34 | 22 not 33 | 1725 |
| S35 | Limit 34 to (English language and year “2006-current”) | 1284 |
