# Supplementary material for: Brief evidence-based interventions for universal child health services: a restricted evidence assessment of the literature
Source: BMC Public Health. 2020 Jun 24;20:993. doi: 10.1186/s12889-020-09104-7 (PMC7315474; doi:10.1186/s12889-020-09104-7)

Child Social and emotional wellbeing


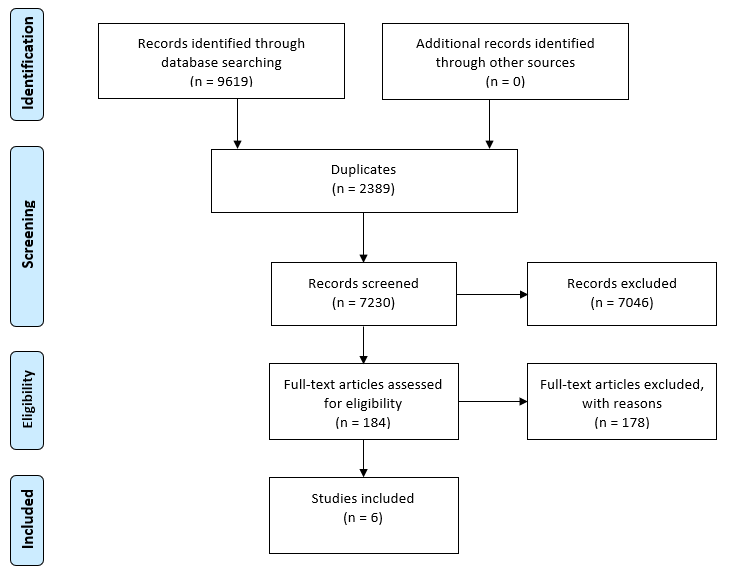


Home learning environment


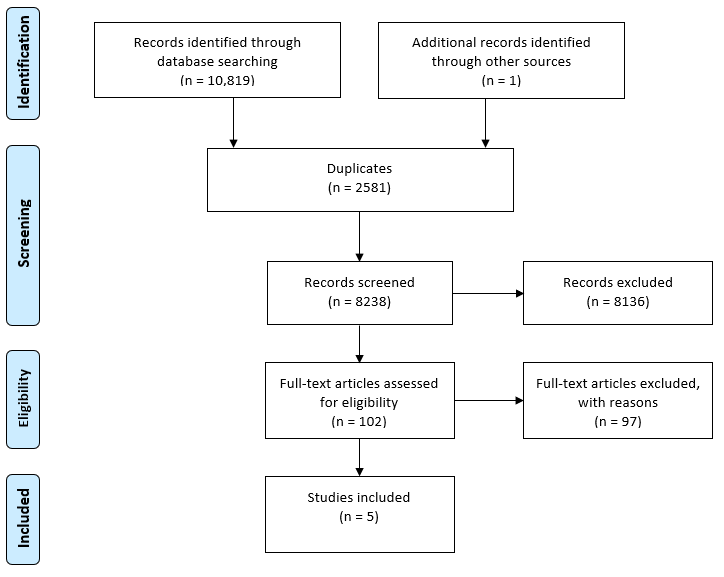


Infant sleep


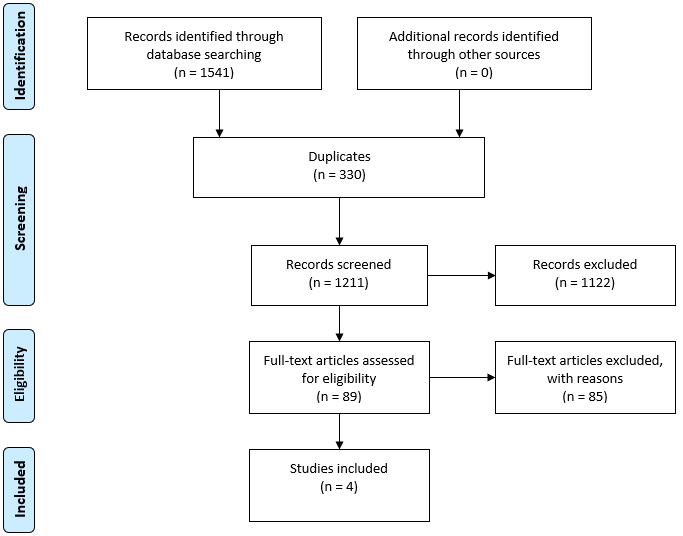


Parent mental health


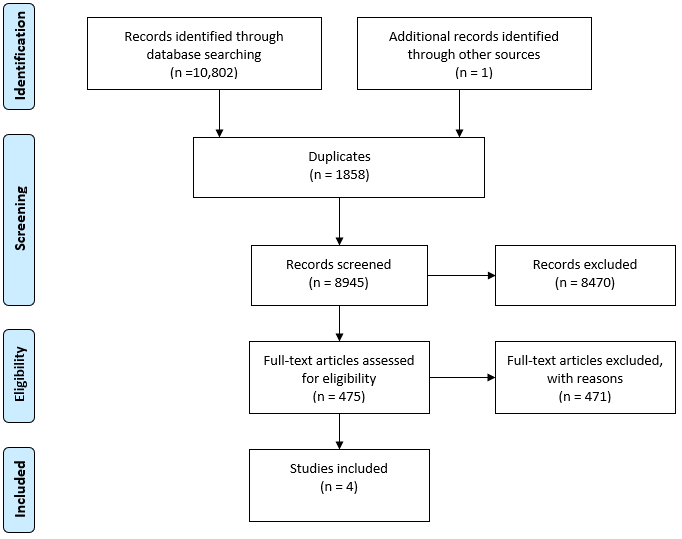

Supplement: Supplementary file 2 — Additional file 2. Prisma flow diagrams (Word document). [file 12889_2020_9104_MOESM2_ESM.docx]
