# Supplementary material for: Brief evidence-based interventions for universal child health services: a restricted evidence assessment of the literature
Source: BMC Public Health. 2020 Jun 24;20:993. doi: 10.1186/s12889-020-09104-7 (PMC7315474; doi:10.1186/s12889-020-09104-7)
Supplement: Supplementary file 3 — Additional file 3. Table of individual studies (Word document). [file 12889_2020_9104_MOESM3_ESM.docx]

Overview of studies included in child SEWB

| **Study,**  **Country, Approach, Design** | **Sample** | **Setting & Delivery** | **Intervention Details** | **Outcome** | **Comments** |
| --- | --- | --- | --- | --- | --- |
| Shaw  (2006) [38]  USA  Selected/  Indicated  RCT | **Recruitment setting**  Families attending income-eligible service invited to complete a questionnaire about child’s disruptive behaviour/emotionality, parenting hassles and maternal depression to determine the presence of socioeconomic, family, and/or child risk factors. Those with risk factors were invited to take part in the RCT.  **Eligibility criteria:** Male infants, aged ~2 years | **Setting**  Participants’ homes  **Who delivered the intervention?**  Master’s level therapist and a master’s level social worker  **Outcome measures**  Child Behavior Check List (CBCL)  Home Observation for Measurement of  the Environment (HOME) | **Intervention** - “Family Check Up” (FCU) takes an ecological and motivational approach by broad assessment of the family context and parenting practices through individual home visits and providing feedback tailored to each family’s needs based on the assessment and the parents’ motivation to change. Motivational interviewing is used to support parents to change problematic parenting practices and identify appropriate support services.  **Length** - 3 intervention sessions  **Follow up period** - 2 years  **Control group-** Usual care | Children in the intervention group showed significantly greater reductions in disruptive behaviour between the ages of 2 and 3 years when compared to controls. Some benefit was sustained until age 4, albeit to a lesser degree.  Greater intervention effects in children with high levels of problem behaviour and conduct problems at baseline.  Significant increase in maternal involvement at 2-year follow up for intervention mothers compared to the control group, while a non-significant decrease was observed in the control mothers. | Of the 60 families assigned to the treatment condition, 55 families (91.7%) participated in sessions.  Participants were reimbursed for completing screening, home visits and assessments.  Dropouts reported significantly higher anxiety and stress scores at baseline and lower efficacy and satisfaction on parenting outcome  Primarily urban area |
| Dishion  (2008) [39]  USA  Selected/  Indicated  RCT | **Recruitment setting**  Reported for Shaw (2006)  **Eligibility criteria**  Aged ~2 years | **Setting**  Participants’ homes  **Who delivered the intervention?**  PhD and masters level service workers  **Outcome measures**  Child Behavior  Checklist (CBCL)  Center for Epidemiological Studies on Depression Scale (CES-D)  Coding of videotaped parent – child interactions | **Intervention**  Reported for Shaw (2006)  **Length** - 3 intervention sessions  **Follow up period** - 2 years  **Control group-** Usual care | Caregiver reports at child ages 2, 3, and 4 years revealed decreased behaviour problems when compared with the control group.  The reductions in child behaviour problems occurred predominantly among families reporting high levels of problem behaviour at baseline.  Intervention mothers reported a significantly greater decrease in depressive symptoms than control mothers at one year follow up; and these changes mediated the intervention effects on child problem behaviours at 2-year follow up | No difference in retention rates between intervention & controls  Of the families assigned to the treatment condition, 77.9% participated in the initial interview and feedback sessions at child age 2, and 65.4% participated at child age 3. Of those families who met with a parent consultant, the average number of sessions per family was 3.32 (SD=2.84). The number of sessions was uncorrelated  with future levels of problem behavior after controlling  for initial levels.  Groups did not differ by child gender or ethnicity, maternal depressive symptoms, or any type of child problem at baseline nor follow up.  Participants were reimbursed for completing assessments. |
| Hayes (2008) [40]  Australia  Indicated  RCT | **Recruitment setting**  Mothers who had self-referred to health centre  after experiencing difficulty managing their infants or  toddlers.  **Eligibility criteria**  Sufficient English language | **Setting**  Health centre  **Who delivered the intervention?**  1 maternal and child  health nurse and 2 early childhood workers.  **Outcome measures**  Parenting Sense of Competence Scale  Difficult Behavior Assessment Form  Depression Anxiety Stress Scale | Mothers attended an intensive, six hour, skills based day stay service to improve infant and toddler care and reduce parental distress. Parents received an individual care plan covering a range of topics and participated in group and individual programs throughout the day.  **Length –** 1 session, 6 hours  **Follow up period-** 6 weeks post intervention  **Control group-** Enhanced waitlist control | Parents from the intervention group reported significant reductions in the frequency and severity of problem child behaviours, mood (depression, stress and anxiety) and parental sense of competency and satisfaction post-intervention; and effects remained 6 weeks follow up. No differences were observed in the control over the same period on any of the measures.  After receiving the intervention, the control group displayed the same significant improvements for problem child behaviours and parental sense of competency and satisfaction but did not show significant reductions in stress and anxiety measures. | No significant difference between the intervention and waitlist groups on demographic data nor outcome measures.  Up to 50% no longer required the service when they were finally offered a place. The high nonparticipation rate is due to the center’s telephone triage process. The triage service includes: (a) instructional advice for managing the presenting problem, (b) allows mothers to call triage if more advice is needed while they are on the waitlist, (c) advises mothers of other services, and (d) recommends that mothers should book into more than one early parenting center and that they should accept the first available place. |
| Hiscock  (2008) [41]  Australia  Universal  Cluster RCT | **Recruitment setting**  Mothers receiving routine health visits  **Eligibility criteria**  Sufficient English language  Infants ~6-7 months old | **Setting**  Health centre  **Who delivered the intervention?**  A nurse and co-facilitator expert in running parenting programs.  **Outcome measures**  Parent behavior checklist  Child Behavior  Checklist (CBCL)  Depression Anxiety Stress Scale | Mothers in the intervention group completed the “Toddlers without Tears” program. At child age 8 months mothers received 4 educational handouts discussing child behaviour and development. Mothers then attended two, 2-hour group education sessions at child ages 12 and 15 months. Sessions covered key modifiable parenting risk factors for childhood behavioural problems: unreasonable expectations, harsh parenting, and lack of nurturing parenting. The program aimed to increase desired behaviour and to reduce unwanted behaviour.  **Length –** 2 sessions, 4 hours  **Follow up period -** 3 months post-intervention (child age 18 months) & 9 months post-intervention (child age 24 months)  **Control group -** Usual care | No significant difference in externalising and internalising infant behaviours or maternal mood (depression, anxiety and stress) was observed at 18 or 24 months follow up (3 and 9 months post-intervention, respectively).  Parenting behaviour domains showed no significant difference at 18 months, although trends were shown for reduced ‘unreasonable expectations’ and ‘harsh discipline’ in intervention mothers compared to control mothers. By 24 months, these differences became significant but there remained no difference in nurturing behaviour.  A follow up of families with the infants at 3 years (21 months post-intervention) found that only the significant difference in ‘unreasonable expectations’ remained. | Non-participating families were more likely than participating families to have low index of disadvantage scores – although the difference was only marginal.  Families who completed the follow-up questionnaires were representative of the baseline sample at all follow up timepoints with respect to sex and temperament of children, maternal education and mental health, and household income and index of disadvantage score.  On average, the number of parents at each group session  was seven (range 2-12).  In the intervention arm, 67% families attended a 12-month group session and 56% attended a 15-month group session. 49% attended both group sessions |
| Dittman  (2015) [42]  New Zealand & Australia  Indicated  RCT | **Recruitment setting**  Participants were recruited via community and media  outreach or through referral from schools or community agencies in Auckland and Brisbane. Parents who registered their interest for the study participated in a telephone screening interview to assess eligibility.  **Eligibility criteria**  Aged 3-5 years  Parent report of concerns about  child’s disobedient behaviour  Parent was not receiving services for their child’s behavior problems or for their own psychological difficulties  Child did not have a developmental disability. | **Setting**  Unclear  **Who delivered the intervention**  Groups facilitated by a registered psychologist  **Outcome measures**  Eyberg Child Behaviour Inventory (ECBI)  30-item Parenting Scale (PS)  Parenting Task Checklist (PTC)  Depression Anxiety Stress Scales (DASS)  Parents in cohabiting relationship: Parent Problem Checklist (PCC)  Relationship Quality Inventory (RQI) | **Intervention**  Preventively-focused discussion group Children did not attend the session. Parents encouraged to share their own experiences with disobedient behaviour and participated in exercises that helped them apply the ideas and strategies presented to their own situation. Parents were taught about reasons for disobedience, including the role of parent behavior in reinforcing noncompliant behavior, the use of positive praise and attention to motivate children to show desirable behavior, and the use of consistent, assertive discipline techniques to manage disobedient behavior and promote compliance. Given parenting plan checklist to use at home to support strategy implementation. Parents received a workbook that contained the content and exercises covered during the group, as well as a parent tip sheet on managing disobedient behavior.  **Length**  One session  **Follow up period**  4 weeks after they attended the Dealing with Disobedience discussion group.  **Control group**  Waitlist | Parents in the intervention group reported greater improvements in disruptive child behavior, ineffective parenting practices and parenting confidence, as well as clinically significant improvements in child behavior and parenting.  No group differences were found for parental wellbeing, inter-parental conflict and general relationship quality. | There were minimal missing data at each time point (<5 %).  Control group had significantly higher Parenting scale Laxness scores at baseline  There were no other significant differences in demographic characteristics or baseline scores between intervention groups, trial sites or between non-completers and completers  Despite the ethnic and economic diversity of the communities from which the participants were recruited, participants were relatively homogenous |
| Hiscock (2018) [43]  Australia  Universal/  Indicated  3 arm RCT | **Recruitment setting**  Mothers receiving routine health visits  **Eligibility criteria**  All mothers attending 8 month visit. Excluded children with a major medical condition and caregivers unable to complete English surveys | **Setting**  Health centres  **Who delivered the intervention**  Provisional psychologists (Family Check up).  Nurse & parenting expert (Toddlers without tears)  **Outcome measures**  Parent behavior checklist  Child Behavior  Checklist (CBCL)  Depression Anxiety Stress Scale  Assessment of Quality of Life 6D  PEDSL | **Interventions**  Targeted  Participants deemed ‘at risk’ were invited to receive the Family Check Up intervention (Reported for Shaw 2006)  Combined  All participants received the Toddlers Without Tears program (Reported for Hiscock 2008) and if they were deemed at risk they also received the Family Check up).  **Length**  Targeted: 3 individual sessions  Combined: 3 group sessions and potential of 3 individual sessions  **Follow up period**  4.5 years  **Control group**  Usual care | At 3 and 4.5 years, there were no significant differences for externalizing or internalizing behavior problems. At 3 years, primary and secondary caregivers reported less over-involved/ protective parenting in both the combined and targeted versus usual care arm; secondary caregivers also reported less harsh discipline in the combined and targeted versus usual care arm.  No significant difference in parental mental health or child quality of life. | At both time points, primary caregivers who were followed up were more likely to have completed high school than nonresponders. Primary caregivers who completed the 3-year follow-up were more likely to come from a higher socioeconomic area than those who did not. Socioeconomic status was similar between the 2 groups among those completing the 4.5-year follow-up than non-responders.  Most parents were married, spoke English, had finished formal schooling, and were not experiencing severe disadvantage.  For the purposes of the narrative synthesis only the findings from the targeted group were used as those at risk in the combined group would have had by default up to 6 sessions with the potential of 4 additional sessions. Reviewers agreed this would exceed the definition of a ‘brief intervention’. |

Overview of studies included in sleep interventions

| **Author, Year, Country** | **Sample** | **Setting & Delivery** | **Intervention Details** | **Outcomes** | **Comments** |
| --- | --- | --- | --- | --- | --- |
| Gradisar  (2016) [44]  Australia  Indicated  RCT | **Recruitment setting**  Parents contacted researchers  in response to adverts. Parents completed a questionnaire and interview  assessing infant’s medical and sleep history.  **Eligibility criteria**  *Inclusion*: ≥1 parent identifying their child having a “sleep problem” & health check ≤1 month where infant was healthy and attained expected weight gain; and typical infant development  *Exclusion:* Edinburgh Postnatal Depression Scale scores ≥15, and indication of suicidality | **Setting**  Participants’ homes  **Who delivered the intervention?**  Research staff members  **Outcome measures**  Coding of videotaped parent – child interactions  Sleep diary  Child Behavior Checklist  Depression and stress  subscales of the Depression Anxiety  Stress Scale–Short Form | Two intervention arms: Both groups met with the researcher once to learn how to implement their allocated behavioural intervention. Parents were provided with an information booklet on the intervention and 24/7 telephone support.  **Graduated extinction**: set schedule of gradually delaying parents’ response to their infant’s cry. Parents instructed to put their infant to bed awake and leave the room within 1 minute. When re-entering the room, they comforted their child, but avoided picking the child up and turning the lights on.  **Bedtime fading:** gradually  limiting the infant’s nocturnal  sleep opportunity  **Length -** 1 education session  **Follow up period –** 1 year  **Control group -** Parents provided with sleep information | At 3-month follow-up, large declines in sleep  latency for infants in both intervention groups, but not the control group. Very large decline in the number of awakenings for infants in the graduated extinction group, yet no changes for infants in other groups. Large improvements in wake after sleep onset for infants in the control and bedtime fading groups, and a very large improvement for the graduated extinction group. Total sleep time showed a moderate increase in the graduated extinction and control condition, but little change in the bedtime fading condition.  At 12-month follow-up, no significant differences in emotional/behavioral problems, attachment styles and maternal mood. | Due to ethics committee requirements, parents could swap conditions. This occurred for 2 families (1 from graduated extinction to sleep education control; 1 from sleep education control to graduated extinction).  Most parents were in a marriagelike relationship (92.7%), had higher education qualifications (77.2%), and were middle- to high-income earners. |
| Hiscock  (2007) [45]  Australia  Indicated  Cluster RCT | **Recruitment setting**  Mothers receiving routine health visits  **Eligibility criteria**  Infants born <32 weeks  Aged ~4 months  Sufficient English language  Reporting an infant sleep problem in past 7-months | **Setting**  Health centres  **Who delivered the intervention?**  MCHN  **Outcome measures**  Maternal report of infant sleep  Edinburgh Postnatal Depression Scale  SF-12  Maternal sleep quality and quantity  Global Infant Temperament Scale | Mothers received an individual sleep management plan, information handouts, and implemented a behavioural intervention of choice:  **Controlled crying:** Parents respond to their infant’s cry at increasing time intervals, to allow independent settling  **Camping out:** sitting with their infant until they fell asleep and gradually removing parental presence.  Mothers kept sleep diaries to help recognise sleep patterns/improvements and to help set further goals.  **Length –** 1 nurse consultation  **Follow up period –** 2 years  **Control group -** Usual care | When the infant was aged 10 months and 12 months, intervention mothers reported significantly less infant sleep problems. At 2 year follow up, there was no significant difference between groups in number reporting a sleep problem but significantly less intervention mothers reported sleep problems that persisted throughout the entire study.  Significantly fewer intervention than control mothers reported poor sleep quality and insufficient sleep when the infant was 12 months. No differences were observed when the infant was 10 months.  No significant difference in number of women with clinical levels of depression (EPDS score >9) when the infants was 10 and 12months, but intervention mothers reported significantly lower mean depression and mental health scores at both time points. At 2 year follow up, intervention mothers were signiﬁcantly less likely to report clinical levels of depression symptoms than control mothers and their mean depression scores were also significantly lower.  Neither parenting practices (harsh discipline and nurturing) nor child mental health (externalizing and internalizing behavior problems) differed between the intervention and control groups. | Centres were ranked within each stratum according to the number of infants recruited at 4 months, randomising the largest centre and alternately allocating subsequent ones.  Intervention and control mothers had similar infant, sleep, maternal and sociodemographic characteristics at baseline  There was no factors associated with being lost to follow up in the intervention arm but in the control arm, mothers of female children and those from a disadvantaged background and/or with lower levels of education were disproportionately more likely to be lost to follow-up |
| Adachi (2009) [46]  Japan  Universal  Controlled study (intervention group recruited and then control group recruited) | **Recruitment setting**  Mothers receiving routine health visits  **Eligibility criteria**  Aged ~4 months | **Setting**  Health centres  **Who delivered the intervention?**  Research team  **Outcome measures**  Self-developed questionnaire recording parental behaviours, infant sleep patterns and infant sleep parameters | Received a booklet following the health check-up and group presentation providing guidance on infant  sleep. The booklet provided content regarding the theoretical background and information on reasons for night waking, development of circadian rhythms, optimal environmental conditions and  parental behavioural patterns and strategies for healthy sleep habits.  **Length –** 10 minute group presentation  **Follow up period –** 3 months  **Control group –** Usual care but received  educational booklet after follow-up | Compared to the control group at follow up, "Hold and soothe immediately" was the only undesirable behaviour while “Settle to sleep in the same place” was the only desirable behaviour to be significantly better in the intervention group.  Significant within-group escalation in night waking was observed in the control group but no difference was observed in the intervention group. ‘Difficulty settling’ increased in both groups. | No significant differences in baseline characteristics between groups  No difference in response rates between groups |
| Hiscock (2014) [47]  Australia  Universal  RCT | **Recruitment setting**  Mothers receiving routine health visits  **Eligibility criteria**  Sufficient English language  Infants born<32 weeks  Serious health condition | **Setting**  Health centres  **Who delivered the intervention?**  Nurses and psychologists  **Outcome measures**  Caregiver report of sleep problem, infant day sleep, crying, or  Feeding  Sleep diary  Edinburgh Postnatal Depression Scale  Pittsburgh Sleep Quality Index (parents)  Maternal  Cognitions About Infant Sleep Questionnaire | The intervention including supplying  information about normal infant sleep and cry patterns, settling  techniques, medical causes of crying and parent self-care, delivered  via booklet and DVD (at infant age 4 weeks), telephone consultation  (8 weeks), and parent group (13 weeks)  **Length –** telephone consultation and 1.5-hour parent group session  **Follow up period –** 6 months  **Control group – Usual care** | No differences between  groups in caregiver report of infant sleep, crying, or feeding problems at either follow-up.  No group differences in  caregiver reports of depression symptoms at 4 months. However, at 6 months, intervention group caregivers were significantly less likely to have clinical levels of depression (score>9 on EPDS).  Between 4 and 6 months, there was a greater reduction in intervention caregivers’ depression scores (clinical levels and mean scores) compared with control caregivers.  Intervention caregivers also had fewer doubts of their ability to manage their infant’s sleep at both time points. At 6 months, intervention caregivers  reported less difficulty setting limits and less excessive concern about sudden infant death.  At 4 months, intervention caregivers had sought help more often from health professionals for their infant than control group caregivers, but no difference at 6 months. | Of the intervention group, 92.5% received the telephone consultation and 50.9% attended the group session.  Participating families were more likely than non-participating families to be of higher socio-economic status.  Caregivers who did not complete follow-up were more likely to report a sleep problem at baseline, to have a lower socioeconomic status, to have completed high school, and to speak a language other than English at home.  Caregivers who did not complete sleep diaries sufﬁciently were more likely to be of a lower socioeconomic status, speak a language other than English at home, and to have completed high school or less. |

Overview of studies included in home learning environment

| **Author & Year** | **Sample** | **Setting & Delivery** | **Intervention Details** | **Results** | **Comments** |
| --- | --- | --- | --- | --- | --- |
| O’Hare & Connolly (2010) [50]  N.Ireland  Universal  RCT | **Recruitment setting**  Mothers receiving routine health visits  **Eligibility criteria**  Age~2 years old | **Setting**  Health centres  **Who delivered the program?**  Health visitors  **Outcome measures**  Parental attitudes to:   - reading and books - sharing/reading books with their child   Family use of libraries | The Bookstart+ intervention involved the delivery of a pack of books and associated reading materials by a health visitor during the standard visit to families with a 2-year-old child, providing a demonstration and encouragement to parents in how to share books, stories, and rhymes with their child.  **Length -** 1 intervention session for book provision and program explanation  **Follow up period –** 3 months  **Control group -** Usual care | The program had a significant positive benefit on parent’s attitudes to reading and books but did not affect parental attitudes to sharing and reading books with their child, and family use of the library.  Subgroup analysis comparing the outcomes across participants with varying socioeconomic background, number of children in the family, and geographic area found no significant differences. | There were no differences between the two groups at baseline and they remained well matched with no significant differences arising due to the levels of attrition. |
| Goldfeld (2012) [49]  Australia  Universal  Cluster RCT | **Recruitment setting**  Mothers receiving routine health visits  **Eligibility criteria**  Sufficient English language | **Setting**  Health centres  **Who delivered the program?**  MCHNs  **Outcome measures**  Sutherland Phonological Awareness  Test Revised  Clinical Evaluation of Language  Fundamentals– Preschool, Australian Second Edition  StimQ-P  Matrices subtest of the Kaufman Brief Intelligence Test, Second Edition  Word reading subtest of the  Wide Range Achievement Test  Adult Test of Non-Word Repetition  Mill Hill Vocabulary Scale | At each time point, intervention nurses spent 5 minutes delivering, modelling, and discussing the Let’s Read literacy promotion messages with the parent. Each intervention family also received a Let’s Read take-home pack containing an age-appropriate picture book, book list, and guidance materials designed to enhance literacy acquisition through shared reading activities characterized by interactive reading style, parental verbal responsiveness, and appropriate book selection.  **Length -** Program was delivered at 4, 12, 18 and 42 months during universal well child visits  **Follow up period –** 4 years  **Control group -** Usual care | No significant difference was observed between groups in measures of child literacy, child language, home literacy environment, child nonverbal cognitive ability, parent language abilities and parental attitudes to reading. | Intervention infants were on average slightly older at recruitment, with slightly more boys and Australian-born parents.  Compared with demographic characteristics of the local areas, the study sample had a slightly higher proportion of primary caregivers who had completed high school , were born in Australia, and who mainly spoke English at home (87.1% vs 68.3%) |
| Wu (2012) [51]  Taiwan  Universal  Controlled study | **Recruitment setting**  Mothers receiving routine health visits  **Eligibility criteria**  Aged ~4-16 months | **Setting**  Health centres  **Who delivered the program?**  Paediatrician and trained volunteers  **Outcome measures**  Adult literacy (caregiver’s reported reading habit, education level)  Home literacy (frequency of book sharing, reading as one of the three favorite interaction activities).  Summation scores of frequency of book-sharing, children’s interest in book-sharing and reading as one of the three favourite interaction activities was coded as child-centered  literacy score (CCLS) | Reach Out and Read: intervention program with 3 components: 1) volunteers reading in the waiting room,  2) age-appropriate book distribution at the clinic, 3) literacy anticipatory guidance and handout, counselling by the paediatrician at enrolment and at each later well-baby clinic (4-18 month scheduled vaccination).  **Length -** 3 sessions between child age 4 and 18 months  **Follow up period -** ~12 months  **Control group -** Usual care | The intervention group exhibited significantly greater increase in child-centered literacy scores from baseline to post intervention, and effects were shown irrespective of the child’s age at baseline. | Control group recruited from general paedatric clinic at comparable age.  No differences in baseline demographics. |
| Christakis (2007) [48]  USA  Universal  RCT | **Recruitment setting**  Mothers receiving routine health visits  **Eligibility criteria**  Aged ~1.5 to 2.5 years  Sufficient English language | **Setting**  Participant homes  **Who delivered the program?**  Parents were mailed resources and provided them to their child  **Outcome measures**  MacArthur-Bates Communicative Development Inventories  Child Behavior Checklist – Hyperactivity subscale | The intervention group received building blocks and parents received 2 newsletters with “blocktivities,” which were suggestions of things that they could do with their child and the blocks (sort them by colour, see how big a stack they could make, etc.). 2 months later a second set of blocks were sent.  **Length -** Set 1 was mailed one week post study enrolment and set 2 was mailed 2 months post enrolment  **Follow up period -** 6 months  **Control group -** Waitlist control | The intervention was associated with significantly higher language scores in a sample of middle- and low-income children but there was no significant effect on attention problems. | Did not report the number of drop outs per intervention group.  Data on parents who refused to participate were not available.  Of the 175 enrollees, at least 1 diary was returned from 92 (53%).  No difference in follow-up rates of diary completion between study arms. |
| Shah (2018) [52] | **Recruitment Setting**  Waiting area of primary care clinic  **Inclusion criteria** Caregiver of a child between 2 and 6 months of age; at least 18 years of age; at child scheduled for a well-child visit. Excluded if child being seen for a sick visit; could not communicate in English. | **Setting**  Waiting area of primary care clinic  **Who delivered the intervention?**  Ancillary staff (hospital volunteers, community health workers)  **Outcome measure**  StimQ-Infant sub-scales: Availability of Learning Materials (ALM) to assess children’s access to toys or other learning materials provided by the caregiver; (2) Parental Involvement in Developmental Advance (PIDA) to assess parent playing and teaching activities that promote children’s cognitive development; and (3) Parental Verbal Responsivity (PVR) to assess parents’ verbal interactions and responsivity to their child.  Parenting Sense of Competence Scale (PSOC) | **Intervention**  Sit Down and Play (SDP) incorporates key constructs of social cognitive theory to encourage positive parenting behaviors through take-home play activities. Staff: (1) model examples of how to use simple age-specific toys to facilitate talking and playing with a child; (2) engage caregivers in discussions regarding their child’s current developmental abilities and the importance of talking, playing, and interacting with their child; (3) observe caregivers using the toy to play with their child and provide feedback, which emphasizes praising and reinforcing positive behaviors; (4) give the caregiver the toy to take home with a handout containing suggestions for other simple play activities to do at home; and (5) encourage caregivers to incorporate playtime with their child as often as possible and provide suggestions on how to integrate play into daily activities such as diaper change and meal times to reinforce the importance of frequent parent–child play on their child’s development.  **Length –** 2 10-minute individualised session  **Follow up period** 1 month  **Control group -** Center for Disease Control and Prevention (CDC) handouts that provided information regarding children’s development, including social-emotional, cognitive, language, and communication milestones. | A significant main effect of time, and as hypothesized, an interaction between time and condition emerged that favored SDP on play behaviors. Post-intervention, SDP families had significantly higher levels of interactional activities between a parent and child that promote cognitive development.  No significant between-group differences on parenting self-efficacy and confidence emerged. | All participants received a $15 gift card at enrollment and a $25 dollar gift card after completion of the follow-up phone interview.  No significant baseline differences existed between intervention and control families. Of the participants enrolled, most caregivers were women (93%), less than 35 years of age (85%), non-white (88%), and reported incomes less than $50,000 (65%); most children received public health insurance (75%). Seventy-five percent of both intervention and control families attended two well-child visits.  Baseline characteristics of those caregivers lost to follow-up and retained did not statistically differ overall or by study condition. Although couldn’t control for baseline differences in Stim-Q |

Overview of studies included in parent mental health

| **Author & Year** | **Sample** | **Setting & Delivery** | **Intervention details** | **Outcome** | **Comments** |
| --- | --- | --- | --- | --- | --- |
| Fisher (2010) [53]  Australia  Universal  Controlled study (control group recruited and followed up before intervention group was recruited) | **Recruitment setting**  Mothers receiving routine health visits  **Eligibility criteria**  Aged ~1 month  Sufficient English language | **Setting**  Health centres  **Who delivered the intervention?**  3 MCHNs  **Outcome measures**  DSM IV disorder as diagnosed by Composite  International  Diagnostic  Interview (CIDI) | The What Were We Thinking! (WWWT) intervention sessions were organized into two components; ‘About Babies’ and ‘About Mothers and Fathers’. The first covered infant temperament, crying and fussing, recognition of tired cues, sleep needs, establishing feed-play-sleep routines of daily care and settling strategies. The second provided language and strategies to effectively discuss and respond to challenges associated with parenting. E.g. the caregiving workload, changed needs for support and managing the losses and gains associated with parenthood.  **Length -** One half day group session  **Follow up period -** 6 months  **Control group -** Standard primary care | Intervention participants with no reported psychiatric history experienced significantly reduced odds of being diagnosed with a mental disorder at 6-month follow up. However, no difference was observed in those with a psychiatric history at baseline. | Participant were reimbursed with a shopping voucher for completing all interviews.  Women lost to follow up had significantly lower educational attainment, higher self-rated confidence on discharge from maternity hospital and reported fewer breastfeeding problems at baseline. Intervention mothers were significantly older, more likely to speak English at home, to have completed post-secondary education, to be in professional or managerial employment and to be multigravid, and less likely to report that the inpregnancy had been unintended than women in the control group. Babies in the intervention group were more likely to be breastfed and cried and fussed for longer periods than those in the control group  63.5% of intervention mothers attended the program and received a folder of written materials for take-home reference. The folder of written materials was posted to all those who did not attend in person.  No significant between-group differences in number/severity of coincidental adverse events experienced during the study. |
| Giallo (2014) [54]  Australia  Universal  RCT | **Recruitment setting**  Mothers receiving routine health visits  **Eligibility criteria**  Sufficient English language | **Setting**  Patients homes  **Who delivered the intervention?**  MCHN  **Outcome measures**  Fatigue Assessment Scale  Fatigue Severity Scale  Depression, Anxiety and Stress Scale | Wide Awake Parenting (WAP) aimed to decrease postnatal fatigue. Strategies focused on how to use energy more efficiently, how to recharge and replenish energy through self-care, improve diet and exercise, manage expectations and establish a healthy sleep routine. Participants were allocated to one delivery method:  **Self-directed writing intervention**: Received workbook via mail and asked to work through activities at own pace over a 4-week period  **Telephone-support intervention**: Received workbook, and phone calls by a health professional to check parents’ understanding of content, answer questions, or guide through intervention.  **Length –** No visit for self-directed writing intervention. 1 home visit and 3 telephone support calls with telephone support.  **Follow up period -** 6 weeks post intervention  **Control group -** Waitlist control | Post-intervention, the telephone-support group reported fewer depressive symptoms than control mothers and fewer anxiety symptoms and stress than the self-directed group. At follow up, only anxiety symptoms differed between the telephone-led and control groups.  Mothers in both intervention groups had significantly higher self-efficacy and intention/  engagement in self-care behaviors than control mothers. The telephone support group reported significantly less perceived barriers to self-care and attributed more importance to self-care than control mothers. Findings were maintained at follow up (apart from perceived barriers only approached significance). | Mothers who withdrew from the study or were lost to follow-up data collection were signiﬁcantly more likely to be born outside Australia, from a non-English speaking background, and report higher levels of anxiety at baseline than mothers who participated in all aspects of the study.  No signiﬁcant differences between the intervention and waitlist control groups.  Treatment adherence data were only completed by 40 (65%) participants in the self-directed written intervention. The majority of participants (75%– 100%) in the telephone-led intervention had read each section, completed the activities, and developed a plan for charging up and saving energy. Although the majority of mothers in the self-directed intervention reported reading the sections of the booklet and completed the activities, approximately one half reported that they had completed their plans for charging up and saving energy. |
| Fisher (2016) [55]  Australia  Universal  Cluster RCT | **Recruitment setting**  Women identified from birth notification lists contacted by telephone  **Eligibility criteria**  Primiparous women <6 weeks postpartum  Sufficient English language | **Setting**  Health centres  **Who delivered the intervention?**  MCHNs  **Outcome measures**  DSM IV disorder as diagnosed by Composite  International  Diagnostic  Interview (CIDI)  PHQ- GAD, Panic, Depression  Barr Parental Diary (Adapted)  Postnatal Attachment Questionnaire  Infant Feeding and Sleeping Arrangements’ Questionnaire | Primiparous women <6 weeks postpartum, their partners and infants, completed the “What Were They Thinking” interactive psychoeducation program, run by a trained MCHN. The program included group discussion, focused tasks, provision of education booklets, short talks and practical demonstrations. The program aimed to prevent common mental disorders.  **Length -** One 6-hour group session  **Follow up period -** 6 months  **Control group -** Usual care | No difference in cases of depression, anxiety or adjustment disorders between groups at follow up.  Lower prevalence of mild to moderate anxiety symptoms and higher self-rated health among those in the intervention group but no difference in depressive symptoms, fatigue assessment measures, prevalence of unsettled infant behaviours or overall satisfaction with the intimate partner relationship.  There was a significantly lower prevalence of the diagnoses in those that received the full intervention compared to the group who received usual care. Receiving only the partial intervention was not associated with a reduction in the primary outcome (may represent an undetected participant bias). | Apart from the seminar, training provided to nurses should enhance quality of care in intervention arm.  Women whose pregnancy was unintended, who had less education and higher baseline IBM Control scores were less likely to provide follow up data but proportion of missing data were comparable.  84% partners attended the group session. The most common reasons given for non-participation were conﬂicting commitments or were unwilling to attend. |
| Glavin  (2010a & b) [56, 57]  Norway  Universal  Controlled study (all well-baby clinics within one municipality received intervention while well-baby clinics in comparison municipality received no intervention) | **Recruitment setting**  Mothers receiving routine health visits  **Eligibility criteria**  Over 18 years old  Sufficient English language  Not currently undergoing treatment for depression | **Setting**  Participants homes  **Who delivered the intervention?**  Public health nurses  **Outcome measures**  Edinburgh Postnatal Depression Scale  Parenting Stress Index | The following elements constituted the main changes of care: (1) a home visit about two weeks after delivery with increased focus on maternal mental health, (2) one supportive counselling session by the PHN after the mothers had completed the EPDS at six weeks postpartum, (3) supportive counselling sessions for the depressed mothers, (4) openness about mental health issues at every visit at the well-baby clinic (5) and a system for referral to further treatment in the municipality. Depending on the women’s needs, women were offered additional counselling until 3 months postpartum. Counselling sessions lasted 30 minutes.  Active listening and emphatic communication (non-directive counselling) were used in the counselling sessions.  **Length –** One session for all. Of those that received additional counselling sessions: range =2–7, mean = 3.4, SD = 1.1  **Follow up period -** 12 months  **Control group –** Usual care | The intervention group reported significantly reduced depressive symptoms at three months (post-intervention) and at six months (follow up). These effects were not influenced by age nor parity and the same pattern of results were shown irrespective of whether the mother had receive a single or multiple session. At one- year follow up, effects were maintained.  Women who had been depressed at least once during the ﬁrst postpartum year reported signiﬁcantly higher levels of parenting stress at 12 months | Non-participating mothers did not differ in mean age or parity from the responders  There were more primiparous women in the intervention group than the control group (52% vs. 34%).  No signiﬁcant differences in age, parity and mean EPDS score at baseline between those who completed the study (responded at all three stages) and those who dropped out before the second or third measurement. |
